# An Unfolded Protein Response Related Signature Could Robustly Predict Survival Outcomes and Closely Correlate With Response to Immunotherapy and Chemotherapy in Bladder Cancer

**DOI:** 10.3389/fmolb.2021.780329

**Published:** 2021-12-23

**Authors:** Facai Zhang, Dechao Feng, Xiaoming Wang, Yiwei Gu, Zhiyong Shen, Yubo Yang, Jiahao Wang, Quliang Zhong, Dengxiong Li, Huan Hu, Ping Han

**Affiliations:** ^1^ Department of Urology/Institute of Urology, West China Hospital, Sichuan University, Sichuan, China; ^2^ Department of Urology, the Affiliated Hospital of Guizhou Medical University, Guizhou, China; ^3^ Department of Urology, the Affiliated Cancer Hospital of Guizhou Medical University, Guizhou, China; ^4^ School of Clinical Medicine, Guizhou Medical University, Guizhou, China; ^5^ Department of Urology, the Second People’s Hospital of Yibin, Sichuan, China

**Keywords:** unfolded protein response, immunotherapy, chemotherapy, bladder cancer, TCGA

## Abstract

**Background:** The unfolded protein response (UPR) plays a significant role in maintaining protein hemostasis in tumor cells, which are crucial for tumor growth, invasion, and resistance to therapy. This study aimed to develop a UPR-related signature and explore its correlation with immunotherapy and chemotherapy in bladder cancer.

**Methods:** The differentially expressed UPR-related genes were put into Lasso regression to screen out prognostic genes, which constituted the UPR signature, and were incorporated into multivariate Cox regression to generate risk scores. Subsequently, the predictive performance of this signature was estimated by receiver operating characteristic (ROC) curves. The CIBERSORTx, the maftool, and Gene set enrichment analysis (GSEA) were applied to explore infiltrated immune cells, tumor mutational burden (TMB), and enriched signaling pathways in both risk groups, respectively. Moreover, The Cancer Immunome Atlas (TCIA) and Genomics of Drug Sensitivity in Cancer (GDSC) databases were used to predict responses to chemotherapy and immunotherapy.

**Results:** Twelve genes constituted the UPR-related signature. Patients with higher risk scores had worse overall survival (OS) in training and three validation sets. The UPR-related signature was closely correlated with clinicopathologic parameters and could serve as an independent prognostic factor. M0 macrophages showed a significantly infiltrated difference in both risk groups. TMB analysis showed that the risk score in the wild type and mutation type of FGFR3 was significantly different. GSEA indicated that the immune-, extracellular matrix-, replication and repair associated pathways belonged to the high risk group and metabolism-related signal pathways were enriched in the low risk group. Prediction of immunotherapy and chemotherapy revealed that patients in the high risk group might benefit from chemotherapy, but had a worse response to immunotherapy. Finally, we constructed a predictive model with age, stage, and UPR-related risk score, which had a robustly predictive performance and was validated in GEO datasets.

**Conclusion:** We successfully constructed and validated a novel UPR-related signature in bladder cancer, which could robustly predict survival outcomes and closely correlate with the response to immunotherapy and chemotherapy in bladder cancer.

## Introduction

Bladder cancer (BC) is a common malignancy in the urinary system, with an estimated 500,000 new cases diagnosed and 200,000 deaths worldwide each year (Antoni et al., 2017; Lenis et al., 2020). It includes a spectra of diseases, including non-muscle-invasive bladder cancer (NMIBC), which is characterized by easy recurrence to aggressive muscle-invasive bladder cancer (MIBC), requiring radical cystectomy (Patel et al., 2020; Richters et al., 2020). Although minimally invasive technology, enhanced endoscopy with narrow-band, neoadjuvant chemotherapy, targeted therapy, and immunotherapy have been introduced into clinical practice in recent years, the 5 years survival rates of BC patients have not improved in the past 30 years (Berdik, 2017; Grayson, 2017). Moreover, to predict BC patients’ survival accurately, the AJCC-stage system based on classical pathological parameters also needs to improve (Sun et al., 2020; Zhang et al., 2021a). Therefore, with the widespread application of next generation sequencing (NGS), an increasing number of studies have focused on biomarkers and molecular subtypes of cancers to provide a theoretical basis for the development of new targeted drugs or to more accurately predict the prognosis of patients (Zhang et al., 2021b).

Unfolded protein response (UPR) is a significant signal pathway that surveils the status and fidelity of proteins in the endoplasmic reticulum (ER) and could be activated when unfolded or misfolded protein accumulates in ER (Hetz et al., 2020). UPR restored correct protein conformation and reduced total protein burden in acute and moderate stress, but it could also induce cell apoptosis in chronic or intense stress (Hetz and Papa, 2018; Hetz et al., 2020; Oakes, 2020). Tumor cells often proliferate rapidly and are exposed to intrinsic and external stimuli, which could disrupt the homeostasis of protein and evoke UPR to alleviate endoplasmic reticulum stress (ERS) (Urra et al., 2016; Cubillos-Ruiz et al., 2017; Chen and Cubillos-Ruiz, 2021). Due to the significance of UPR in tumors, many researchers have paid more attention to UPR related biomarkers. It has been reported that OTUB1, P4HB, and EHMT2 could regulate the malignant phenotype of bladder cancer cells via the ERS related URP pathway (Cui et al., 2015; Wang et al., 2020; Zhang et al., 2021c; Wu et al., 2021).

Although several URP-related genes have been identified as biomarkers that influence tumor progression, there are still no UPR-related multigene signatures that can be used to predict patients’ prognosis in BC. To examine this, the present study first constructed and validated a robust UPR-related signature and investigated its correlation with clinicopathological parameters and response to immunotherapy and chemotherapy.

## Materials and Methods

### Data Collection

The RNA-seq and clinical data of bladder cancer patients were downloaded from the TCGA database, which was used as the training set to develop a UPR-related gene signature. Similarly, GSE13507, GSE32548, and GSE48075 in the GEO dataset were retrieved and used as validation sets to validate the UPR-related signature. The UPR-related genes were obtained from hallmark genes from the Molecular Signature database.

### Identification of Differentially Expressed UPR Genes in the TCGA Dataset

We first used the “VennDiagram” package in R software to screen out the co-expressed URP-related genes in the TCGA dataset, GSE13507, GSE32548, and GSE48075. Then, the “limma” package was used to identify the differentially expressed genes between 19 normal samples and 414 tumor samples with a False Discovery Rate (FDR) of **<** 0.05 in the training set.

### Gene Ontology(GO) Enrichment Analysis and Kyoto Encyclopedia of Genes and Genomes (KEGG) Pathway Analysis

All differentially expressed genes were used to explore potentially enriched function and pathways with the “clusterProfiler” package in R software. The enriched KEGG pathways were screened out using *p* < 0.05 and Q < 0.05, and were presented in bubble plots. Similarly, GO terms with *p* < 0.05 and Q < 0.05 were selected and presented respectively according to molecular function (MF), biological process (BP), and cellular component (CC) categories in bar plots.

### Development and Validation of a UPR-Related Signature

All differentially expressed UPR genes were incorporated into the lasso regression model, in which penalties were applied to all differentially expressed UPR genes for preventing the overfitting effects of the model. The penalty parameter (λ) for the model was determined by 10 fold cross-validation following the minimum criteria. After that, the prognostic UPR genes selected by lasso were used to construct the UPR signature and risk scores were generated in a multivariate Cox regression model with the following formula:

$$\text{Risk score}=\overset{n}{\underset{i=1}{\sum }}coefficient_{i}*EXP{\left(mRNA\right)}_{i}$$

According to the median risk score, all patients with different risk scores could be divided into high and low risk groups. Kaplan-Meier analysis was used to compare the survival outcomes of patients in the high and low risk groups in the TCGA database. Patients in GSE13507, GSE32548, and GSE48075 also had risk scores based on the formula above and these three cohorts were used to validate the signature’s prognostic performance.

### Correlation of the UPR Signature With Clinical Parameters

To investigate the relationship between the UPR signature and clinical parameters, patients in the training and validation sets were classified into different subgroups in light of age, gender, T-stage, nodule, metastasis, AJCC-stage, and molecular subtypes, etc, and risk scores in different subgroups were compared. Then, the Kaplan-Meier method was used to explore the survival outcomes of patients with high or low risk scores in different subgroups. Moreover, UPR-related risk scores and these clinicopathological parameters were incorporated into the univariate and multivariate Cox regression model to screen out independent prognostic factors in the training and validation dataset, which were used to constitute a nomogram in the following study. Multivariate ROC curves at different points of time were used to evaluate the predictive performance of the risk score and other clinical parameters.

### Gene Set Enrichment Analysis, Immune Cell Infiltration, and Tumor Mutational Burden Analysis

We uploaded the RNA-seq data of patients in the high and low risk groups to the GSEA website. The differentially enriched pathways in both groups were screened with *p* < 0.05 and FDR<25%. After that, the top enriched pathways in the high- and low risk groups were analyzed and presented in multi-GSEA plots to explore the potential activated pathways in the two risk groups.

Then, the transcriptome data in training and validation sets were normalized and we estimated the contents of 22 immune cells in patients with different risk scores on the CIBERSORTx website. Subsequently, we compared the contents of infiltrated immune cells between the high and low risk groups and presented them in violin plots.

We downloaded the tumor mutational data from the TCGA dataset. The Maftools package in R software was used to analyze these mutational data in both risk groups and TMB was calculated with the tumor specific mutation genes. After that, we listed the top mutational genes in the high and low risk groups individually and compared the risk scores in the wild and mutational groups. A scatter plot was then drawn to explore the correlation between TMB and risk scores. *p* < 0.05 was considered statistically significant.

### Prediction of Immunotherapy Response

As TMB, programmed cell death 1 ligand 1 and 2 (PD-L1 and PD-L2), and microsatellite instable (MSI) associated mismatched repair genes in tumor tissue are believed to be potent biomarkers for predicting immunotherapy response, the transcriptome data of CD274 (PD-L1), PDCD1LG2 (PD-L2), MSH2, MSH6, PMS2, and MLH1 were extracted and compared in both risk groups. Moreover, The Cancer Immunome Atlas (TCIA), a database providing comprehensive immunogenomic analyses based on the TCGA, was used to evaluate the immunotherapy response by generating the immunophenoscore (IPS) in each sample. After that, IPS were compared between the high and the low risk groups. *p* < 0.05 was considered statistically significant.

### Prediction of Chemotherapy Response

Genomics of Drug Sensitivity in Cancer (GDSC), a public pharmacogenomics database, was used to predict the chemotherapy response for bladder cancer patients with different UPR-related risk scores. The chemotherapy response and drug sensitivity were evaluated by the half-maximal inhibitory concentration (IC50) with the “pRRophetic” package in R software, and were compared between the high and low risk groups. *p* < 0.05 was considered statistically significant.

### Development and Validation of a Predictive Nomogram Based on Clinical Parameters and the UPR-Related Risk Score

We selected independent prognostic factors screened by multivariate Cox regression model to establish a nomogram. Age, stage, and UPR-related risk scores were incorporated into the nomogram in the TCGA dataset. An enhanced bootstrap strategy of 1,000 times was used to validate this nomogram internally, while GSE13507 and GSE48075 datasets were applied to validate this model externally. AUR, Brier scores, and calibration plots were used to assess the performance of the nomogram in 1, 3, 5 years.

### Statistical Analysis

All statistical analyses were performed in R software (Version 4.0.5) and GraphPad Prism 9. Quantitative data in two groups were compared using *Student*-t test and quantitative data in three or more groups were compared with one-way analysis of variance (ANOVA) or Welch’s test. *p* < 0.05 was regarded as statistically significant.

## Results

### Selection of UPR-Related Genes and Construction of UPR-Related Signature

**FIGURE 1:**
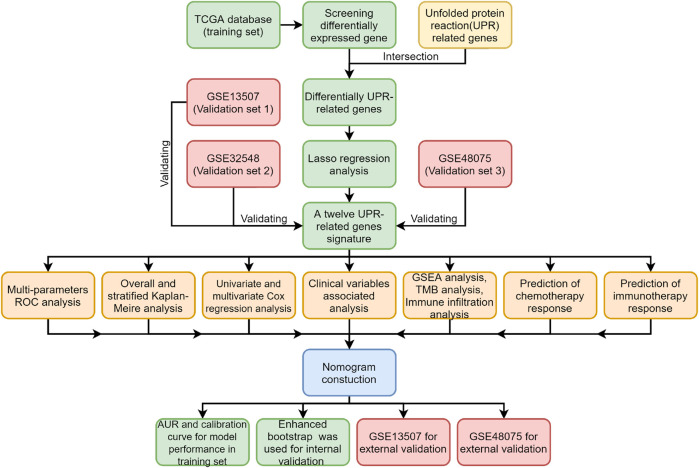
The flowchart of the study.

**FIGURE 2:**
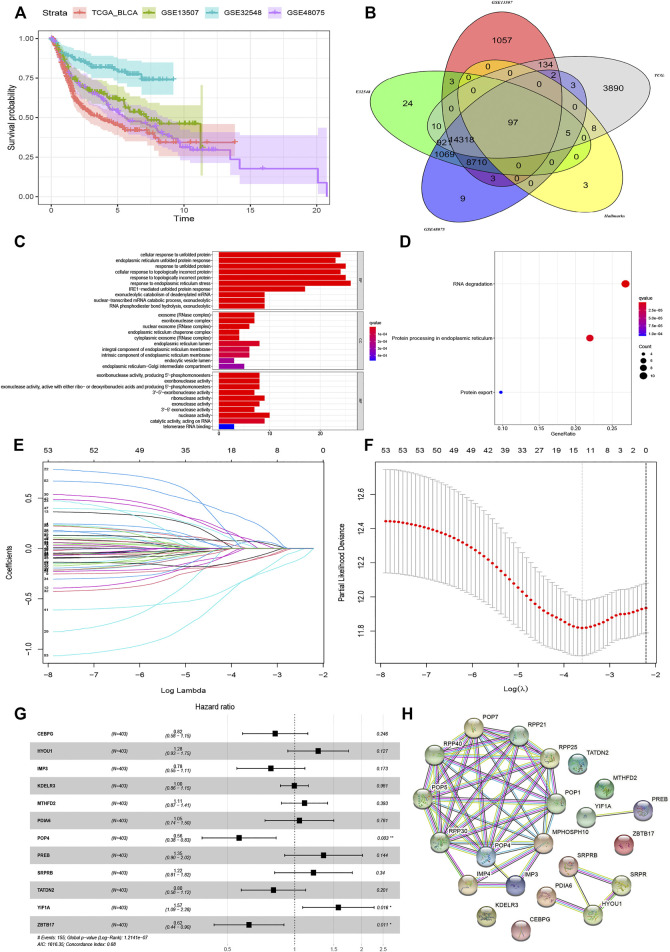
Construction of UPR-related signature utilizing Lasso regression analysis. **(A)** The outcomes variables of TCGA, GSE13507, GSE32548, and GSE48075. **(B)** The Venn diagram showed that 97 UPR genes coexisted in the training and three validation sets. **(C)** GO enrichment analysis revealed that terms of cell response to unfolded protein and endoplasmic reticulum unfolded protein response were significantly enriched, while KEGG enrichment analysis showed that DNA degradation and protein processing in endoplasmic reticulum closely correlated with the differentially expressed UPR-related genes **(D)**. **(E and F)** Differentially expressed UPR-related genes were incorporated into Lasso regression analysis to screen out prognostic UPR genes. **(G)** 12 prognosis related UPR-related genes were incorporated into the multivariate regression model to generate risk scores. **(H)** Protein-protein interaction network was established by STRING with a minimum required interaction score = 0.4.

All procedures and analyses are presented in the flowchart (Figure 1). Table 1 and Figure 2A show the basic characteristics and survival outcomes of the training set and three validation sets. First, we used the Venn diagram to screen out 97 co-expressed genes in the training and validation sets (Figure 2B). After that, the “limma” package in R software was applied to filter out differentially expressed genes among these 97 co-expressed genes between normal samples and tumors with FDR < 0.05 in the TCGA dataset and 53 differentially expressed UPR-related genes were selected. Second, GO and KEGG enrichment analyses were made with these 53 differentially expressed genes and terms such as cell response to unfolded protein, endoplasmic reticulum unfolded protein response were significantly enriched, which were in accordance with the characteristics of UPR-related genes (Figure 2C). Meanwhile, KEGG pathway enrichment analysis showed that those differentially expressed genes closely correlated with RNA degradation and protein processing in the endoplasmic reticulum (Figure 2D). Third, all 53 differential UPR-related genes were incorporated into the lasso regression and 12 prognostic UPR-related genes (CEBPG, HYOU1, IMP3, KDELR3, MTHFD2, PDIA6, POP4, PREB, SRPRB, TATDN2, YIF1A, ZBTB17) were identified and used to construct the UPR-related signature (Figures 2E,F; Supplementary Table S1). Subsequently, we put the 12 prognostic UPR-related genes into the multivariate Cox regression model to generate risk scores according to the formula above (Figure 2G; Supplementary Table S2). Finally, we uploaded these 12 prognostic UPR-related genes to the STRING website and explored the interaction of the potential protein with a minimum required interaction score = 0.4 (Figure 2H).

**TABLE 1:** Baseline clinical characteristics of four databases.

Variables	Number			
	TCGA	GSE13507	GSE32548	GSE48075
Total	408	165	131	131
Age				
<60	87	42	26	32
≥60	321	123	105	99
Gender				
Female	107	30	31	32
Male	301	135	100	99
Grade	10 cases missing		G1+G2 deemed as low grade	
Low Grade	20	105	56	33
High Grade	378	60	75	98
AJCC-stage	2 cases missing	Calculated by 8th AJCC		Calculated by 8th AJCC
0a	0	23	NA	32
0is	0	0	NA	1
I	2	80	NA	27
II	130	26	NA	12
III	140	29	NA	41
IV	134	7	NA	18
T	34 cases missing			
Tis	0	0	0	1
Ta	0	23	40	35
T1	3	81	53	28
T2	119	31	T2+T3+T4 = 38	24
T3	194	19		32
T4	58	11		11
N	42 cases missing			
N0	237	149	NA	106
N1	46	9	NA	20
N2	75	6	NA	5
N3	8	1	NA	0
M	201 cases missing			
M0	196	158	NA	120
M1	11	7	NA	11

NA: not applicable.

### Prognostic Performance of the UPR-Related Signature in the Training Set and Validation Sets

**FIGURE 3:**
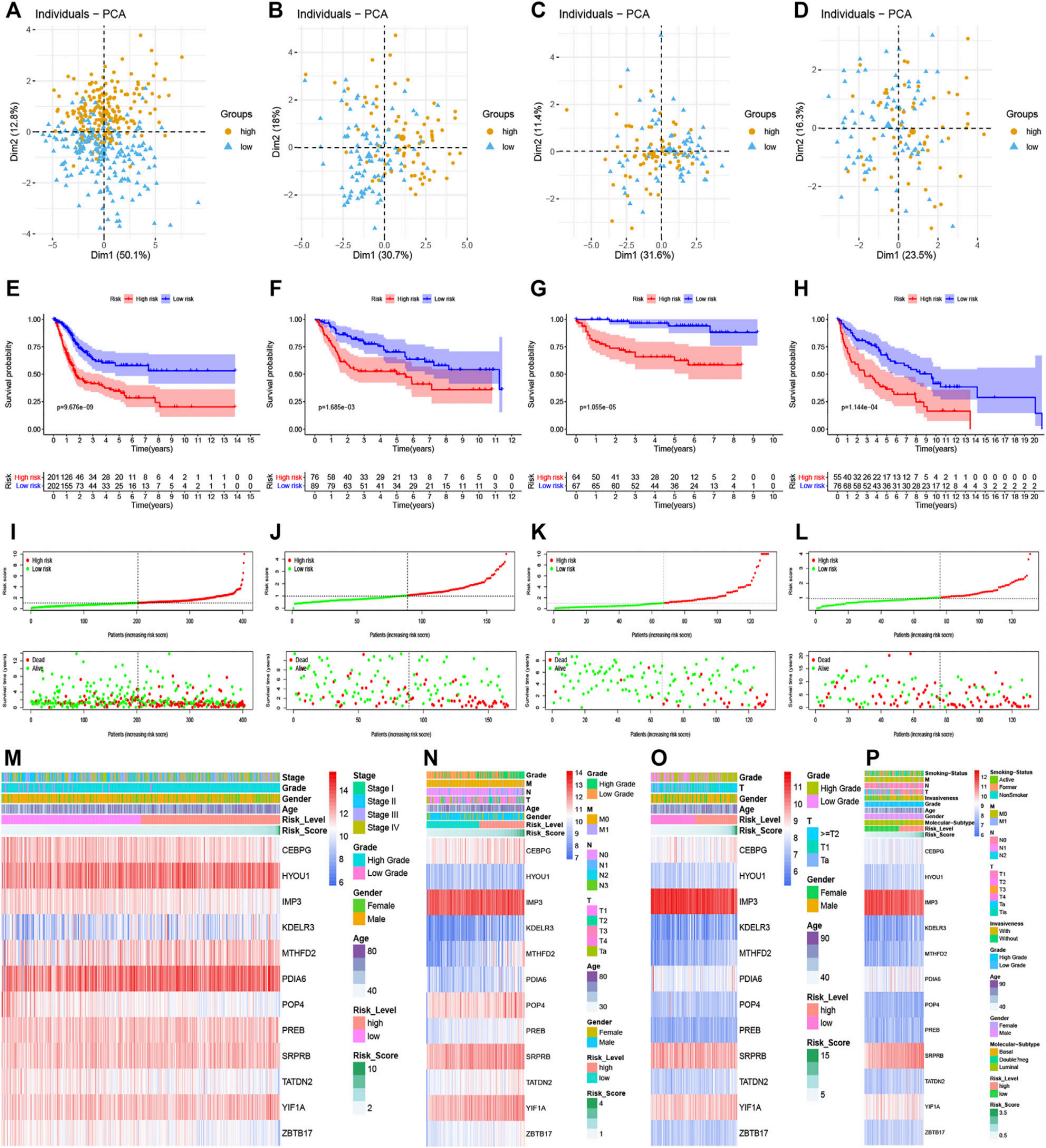
Prognostic performance of the UPR-related signature in the training set and validation sets. **(A–D)** The UPR-related signature could significantly distinguish patients with different risk scores, and patients with higher risk scores had a worse survival time than ones with lower risk scores **(E–H).** **(I–L)** Along with the increment of risk scores, the mortality rates of patients increased, while overall survival of patients was shortened. **(M–P)** Multi-group heatmaps showed the tendency of signature gene expression and clinicopathological parameters in the training and validation sets.

According to the median risk score in the TCGA dataset, all patients with risk scores in the training dataset and 3 validation datasets could be divided into high and low risk groups. As shown in Figures 3A–D, the distribution of patients in the high and low risk groups were quite different in the training and validation datasets, which meant that the risk score had a robust separating capacity. Kaplan-Meier survival curves showed that patients in the high risk group tended to have worse overall survival compared with ones in the low risk group, which were validated by GSE13507, GSE32548, and GSE48075 (Figures 3E–H). Moreover, the scatter points revealed that the mortality of patients increased and survival time decreased along with the increment of risk scores in the training set (Figure 3I), which were also verified by the 3 validation sets (Figures 3J–L). Finally, the multi-group heatmaps were used to present the tendency of signature gene expression and clinicopathological parameters in the training and validation sets (Figures 3M–P).

### Relationship Between the UPR-Related Signature With Clinical Parameters

**FIGURE 4:**
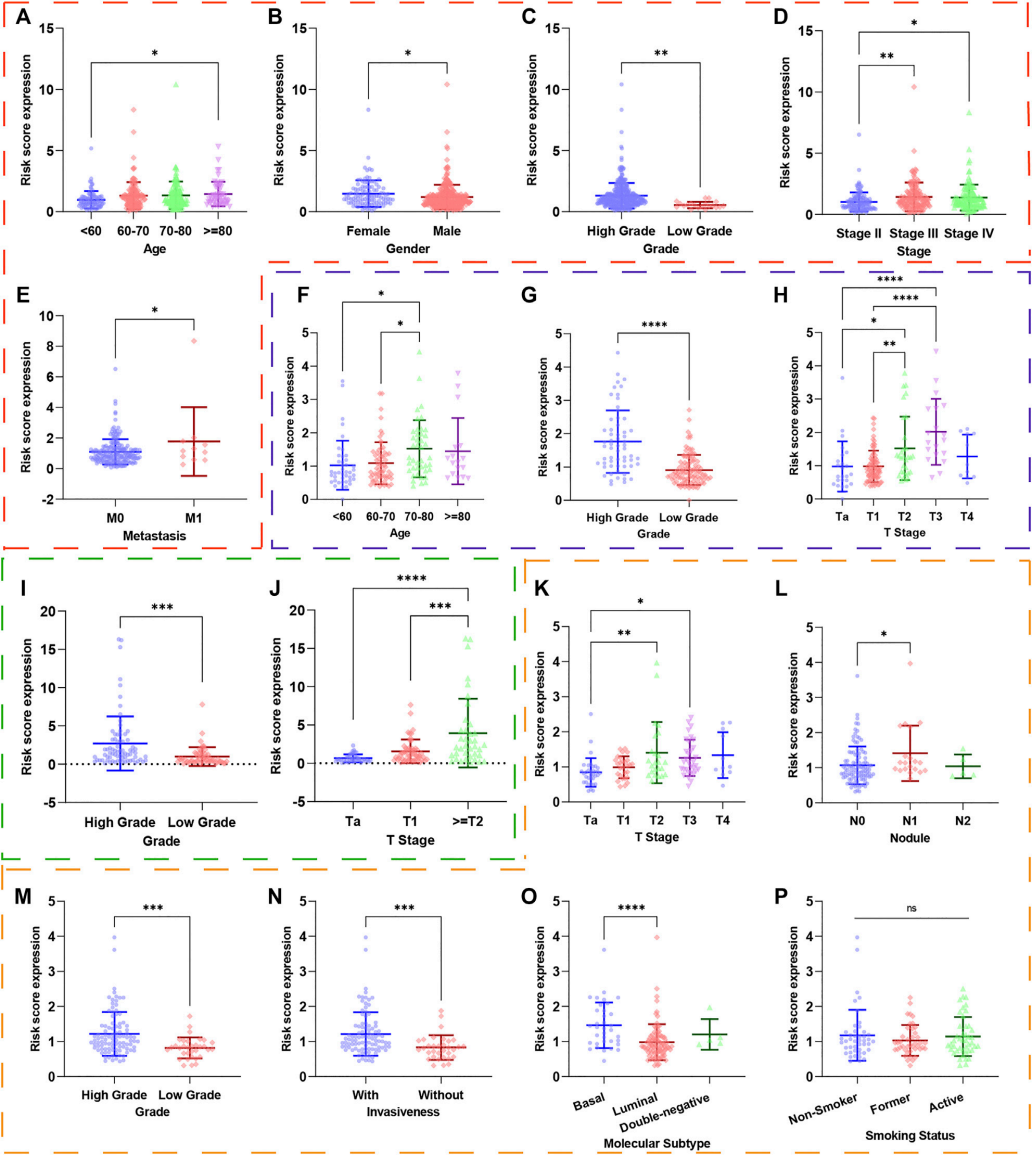
The risk scores of patients in different subgroups were stratified by clinicopathological parameters. The red, blue, green, and orange dashed boxes corresponded to the subgroups in the TCGA, GSE13507, GSE32548, and GSE48075, respectively. The risk scores were significantly different in subgroups of **(A)** age, **(B)** gender, **(C)** pathological grade, **(D)** AJCC-stage, and **(E)** metastasis in the TCGA dataset. Similarly, the risk scores were still different in subgroups of **(F)** age, **(G)** pathological grade, and **(H)** T-stage in GSE13507; in subgroups of **(I)** pathological grade and **(J)** T-stage in GSE32548; in subgroups **(K)** T-stage, **(L)** nodule, **(M)** pathological grade, **(N)** invasiveness, **(O)** molecular subtypes in GSE48075. Interestingly, the risk score did not correlate with smoking (*p*). *p < 0.05; **p < 0.01; ***p < 0.001; ****p < 0.0001; ns: not significance significant.

To investigate the relationship between UPR-related signature and clinical parameters, patients with different risk scores were first stratified by clinicopathologic parameters and risk scores were compared among different subgroups. Patients of older age, with high pathological grade, advanced AJCC-stage, and metastasis tended to have a higher risk score in the TCGA dataset (Figures 4A–E). Similarly, this significant tendency was also validated in GSE13507 (Figures 4F–H), GSE32548 (Figures 4I,J), and GSE48075 (Figures 4K–P), which indicated that the UPR-related signature might be significantly correlated with come classical clinical parameters. It is worth noting that the basal molecular subtype of bladder cancer had a significantly higher risk score compared with the luminal molecular subtypes, which were in accordance with the fact that the luminal subtype of bladder cancer usually had a better prognosis compared with the basal subtype.

**FIGURE 5:**
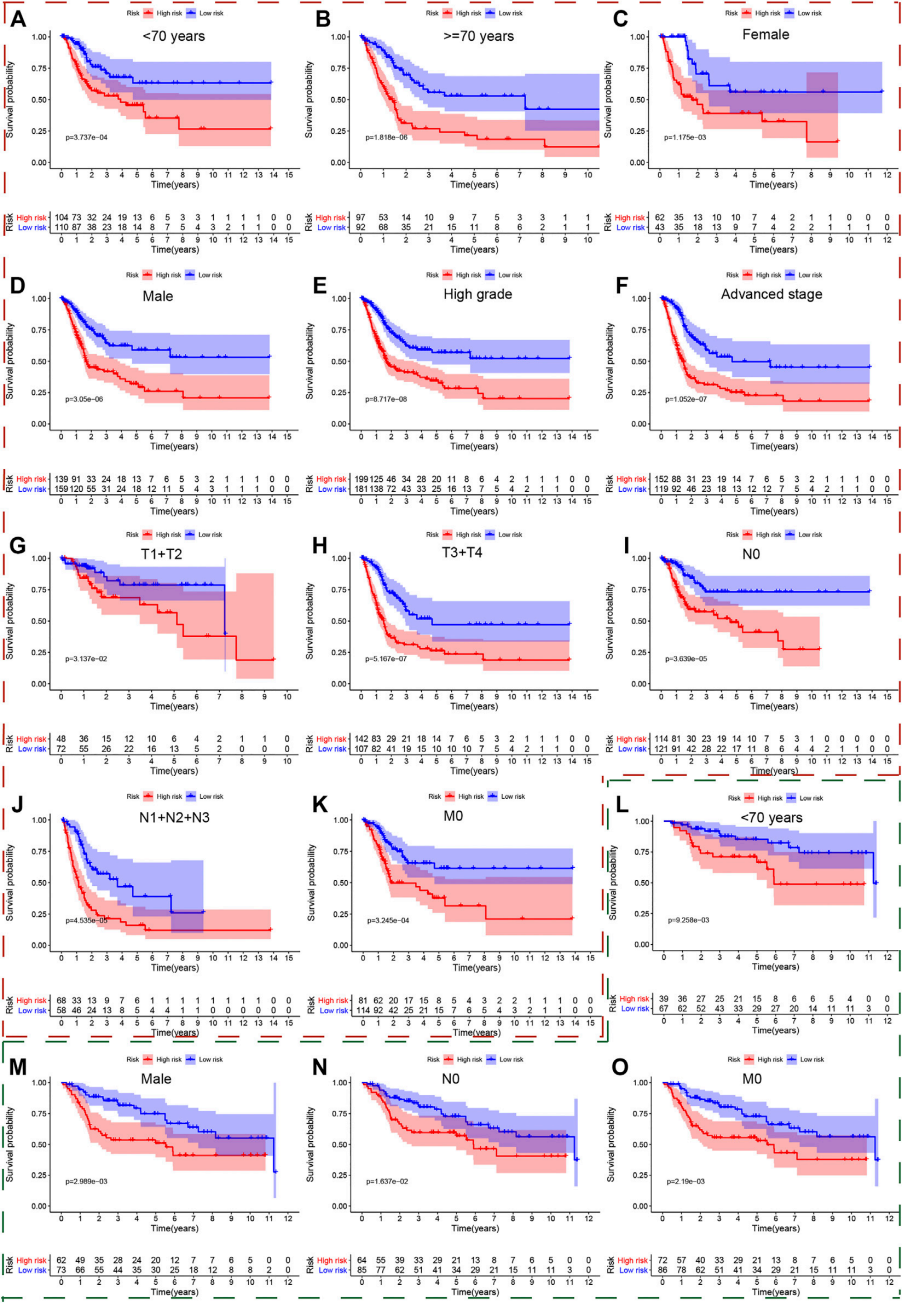
The survival outcomes of bladder cancer patients with different risk scores in subgroups of clinicopathological parameters in the TCGA and GSE13507. The red and green, dashed boxes corresponded to the subgroups in the TCGA and GSE13507, respectively. Patients with higher risk scores had worse overall survival compared with those with lower risk scores in subgroups for **(A)** age < 70, **(B)** age ≥ 70, **(C)** female, **(D)** male, **(E)** high grade, **(F)** advanced-stage, **(G)** low T-stage, **(H)** high T-stage, **(I)** nodal metastasis-free, **(J)** nodal metastasis, and **(K)** metastasis-free in the TCGA; and in the subgroups **(L)** age < 70, **(M)** male, **(N)** nodal metastasis-free, **(O)** metastasis-free in GSE13507.

**FIGURE 6:**
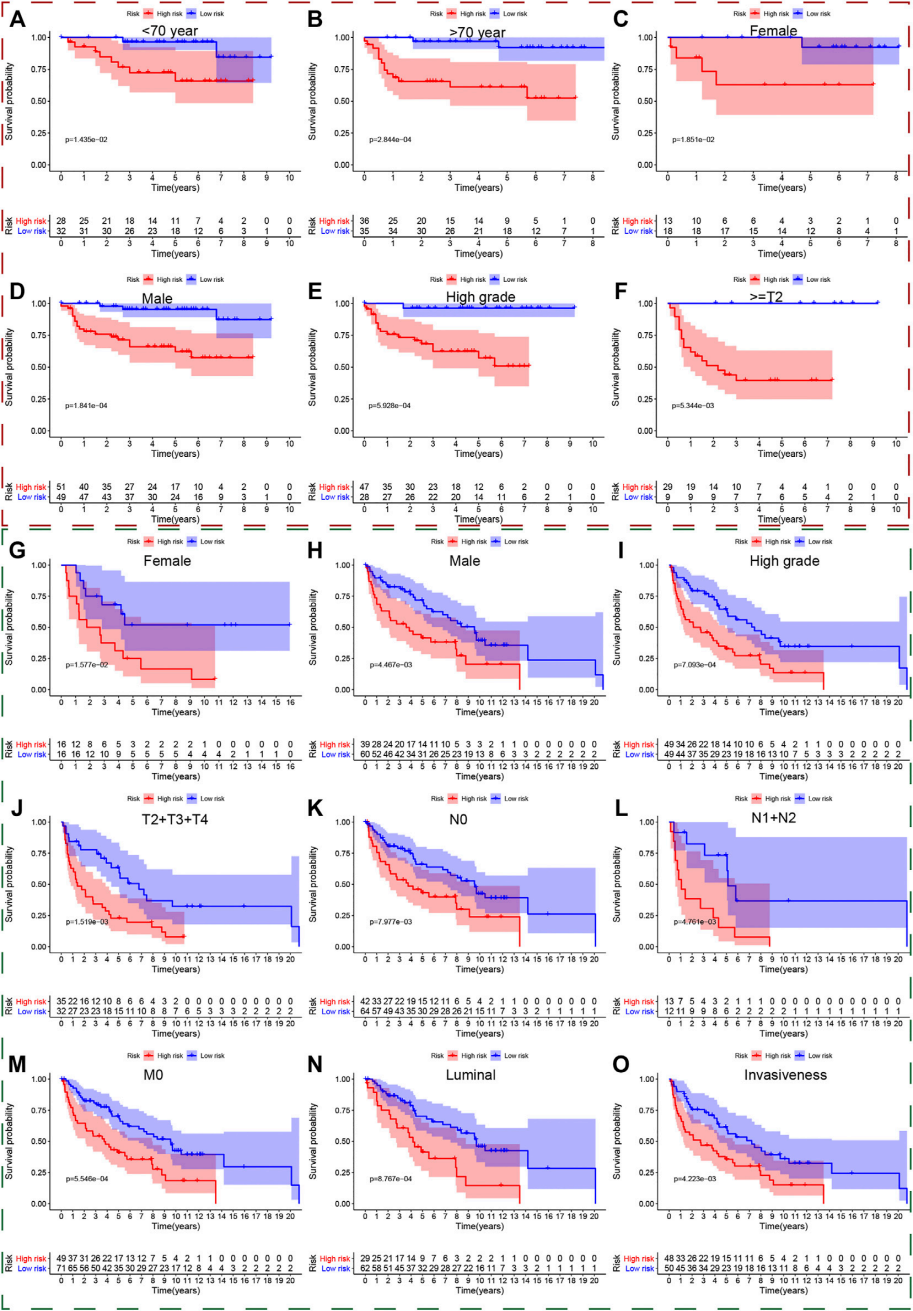
The survival outcomes of bladder cancer patients with different risk scores in subgroups of clinicopathological parameters in GSE32548and GSE48075. The red and green, dashed boxes corresponded to the subgroups in GSE32548 and GSE48075, respectively. Patients with higher risk scores had worse overall survival compared with those with lower risk scores in subgroups of **(A)** age < 70, **(B)** age ≥ 70, **(C)** female, **(D)** male, **(E)** high grade, and **(F)** high T-stage in GSE32548; in the subgroups of **(G)** female, **(H)** male, **(I)** high grade, **(J)** muscle-invasive bladder cancer, **(K)** nodal metastasis-free, **(L)** nodal metastasis, **(M)** metastasis-free, **(N)** luminal subtype, and **(O)** invasiveness in GSE48075.

We also used the Kaplan-Meier method to further analyze the survival outcomes of patients with different risk scores in different subgroups. Patients with higher risk scores had poor survival outcomes compared with ones with lower risk scores in the subgroups of age <70 years (Figure 5A), age ≥ 70 (Figure 5B), female (Figure 5C), male (Figure 5D), high grade (Figure 5E), advanced stage (III + IV) (Figure 5F), low T-stage (T1 + T2) (Figure 5G), high T-stage (T3 + T4) (Figure 5H), nodule-free (Figure 5I), nodule metastasis (Figure 5J), metastasis-free (Figure 5K). Similarly, we also validated the signature’s performance in the subgroups stratified by clinical parameters in GSE13507, GSE32548, and GSE48075. Patients in the high risk group still had significantly worse survival outcomes compared with those in the low risk group in the subgroups of age <70 years (Figure 5L), male (Figure 5M), nodule-free (Figure 5N), and metastasis-free (Figure 5O) in GSE13507. The overall survival time of the low risk group was significantly longer than that of the high risk group in the subgroup of age <70 years (Figure 6A), age >70 years (Figure 6B), female (Figure 6C), male (Figure 6D), high grade (Figure 6E), and high T-stage (≥T2) (Figure 6F) in GSE32548. While the survival difference in both risk groups was still significant in subgroups of female (Figure 6G), male (Figure 6H), high grade (Figure 6I), muscle-invasiveness (T2 + T3 + T4) (Figure 6J), nodule free (Figure 6K), nodule metastasis (Figure 6L), metastasis (Figure 6M), luminal subtype (Figure 6N), and invasiveness (Figure 6F) in GSE48075.

### Selection of Independent Prognostic Factors and Predictive Performance of Clinical Parameters

**FIGURE 7:**
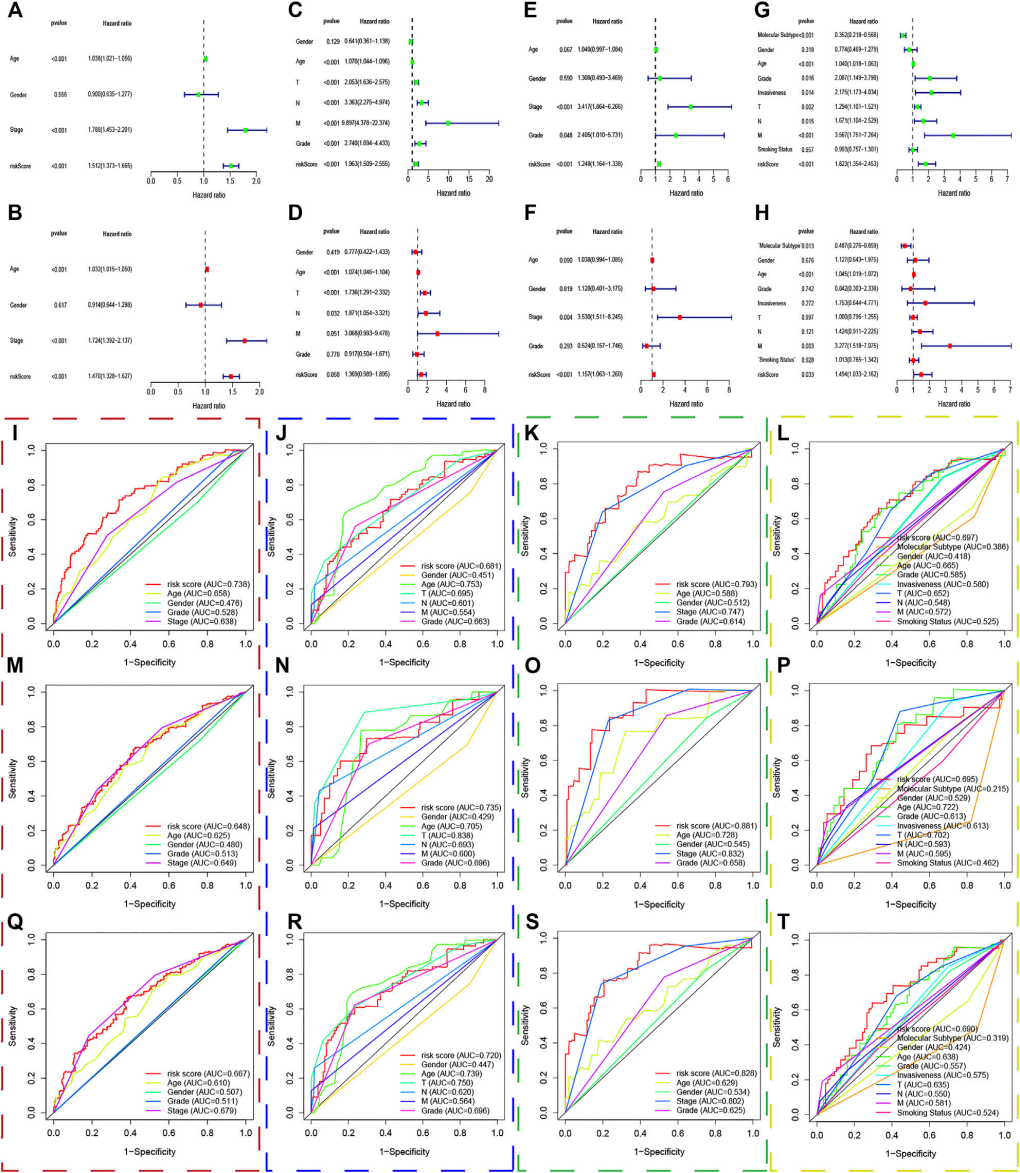
The predictive performance of the risk score and other clinicopathological parameters. **(A–H)** The univariate and multivariate Cox regression suggested that the risk score was an independent prognostic factor in the training and validation sets. The red, blue, green, and yellow dashed boxes corresponded to the TCGA, GSE13507, GSE32548, and GSE48075, respectively. **I**, **J**, **K,** and **L** showed that the 1-year multiple ROC curves of the risk score and other clinicopathological parameters in the training set, GSE13507, GSE32548, and GSE48075, respectively. **M**, **N**, **O,** and **P** showed that the 3-years multiple ROC curves of the risk score and other clinicopathological parameters in the training set, GSE13507, GSE32548, and GSE48075, respectively. **Q**, **R**, **S,** and **T** showed that the 5-years multiple ROC curves of the risk score and other clinicopathological parameters in the training set, GSE13507, GSE32548, and GSE48075, respectively.

We put all the classical clinical parameters and risk scores into the univariate and multivariate Cox regression analysis to screen out independent prognostic factors. Interestingly, the UPR-related risk score was deemed as a significant independent predictor with *p* < 0.05 in the TCGA dataset (Figures 7A,B), GSE32548 (Figures 7E,F), and GSE48075 (Figures 7G,H). Although the risk score cannot be identified as an independent prognostic factor with *p* = 0.058 in GSE13507 (Figures 7C,D), we believed that the small sample size could account for this insignificance to a certain extent.

Multivariate ROC curves were used to evaluate the predictive performance of clinical parameters and the risk score in 1, 3, and 5 years. Unexpectedly, the UPR-related risk scores have the highest discrimination with the highest AUC value compared with traditional clinicopathological parameters used to evaluate tumor progression in the TCGA dataset (Figures 7I,M,Q). Similarly, the discrimination of the UPR-related risk score was validated with a higher AUC value in GSE13507 (Figures 7J,N,R), GSE32548 (Figures 7K,O,S), and GSE48075 (Figures 7L,P,T), which demonstrated the robust predictive performance of the risk score.

### Gene Set Enrichment Analysis

**FIGURE 8:**
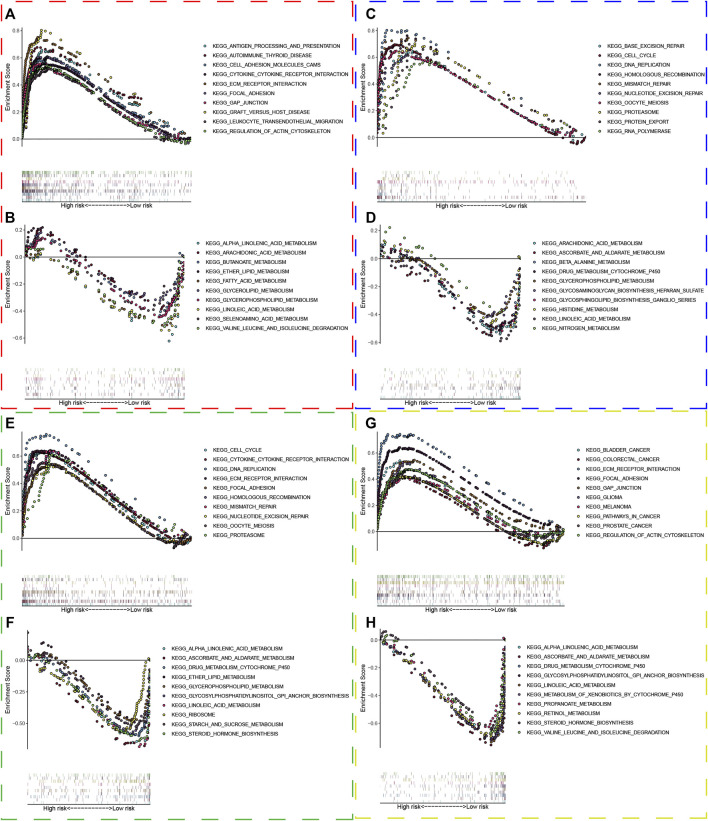
Gene set enrichment analysis. The red, blue, green, and yellow dashed boxes corresponded to the TCGA, GSE13507, GSE32548, and GSE48075, respectively. GSEA results showed significant enrichment of immune-, extracellular matrix-, replication and repair-related signaling pathways in the high-risk group **(A,C,E, and G)**, and significant enrichment of metabolism signaling pathways in the low-risk group **(B,D,F, and H)**.

The transcriptome data of patients in the high and low risk group was uploaded to GSEA, and the top 50 KEGG pathways were enriched with the differentially expressed genes in both risk groups. Interestingly, we found that immune and extracellular matrix associated pathways were enriched in the high risk group in the TCGA dataset, such as antigen processing and presentation, graft versus host disease, ECM receptor interaction, cell adhesion molecules cams, cytokine and cytokine receptor interaction, and so forth. Meanwhile, some metabolism associated pathways belonged to the low risk group, including alpha linolenic acid metabolism, arachidonic acid metabolism, fatty acid metabolism, ether lipid metabolism, and so forth (Figures 8A,B). In the same way, the GSEA analysis showed that replication and repairmen associated pathways were enriched in the high risk groups of GSE13507, GSE32548, and GSE48075 (Figures 8C,E,G), while metabolism associated pathways still belonged to the low risk group, which were in accordance with results of the TCGA dataset (Figures 8D,F,H).

### Immune Cells Infiltration Analysis

**FIGURE 9:**
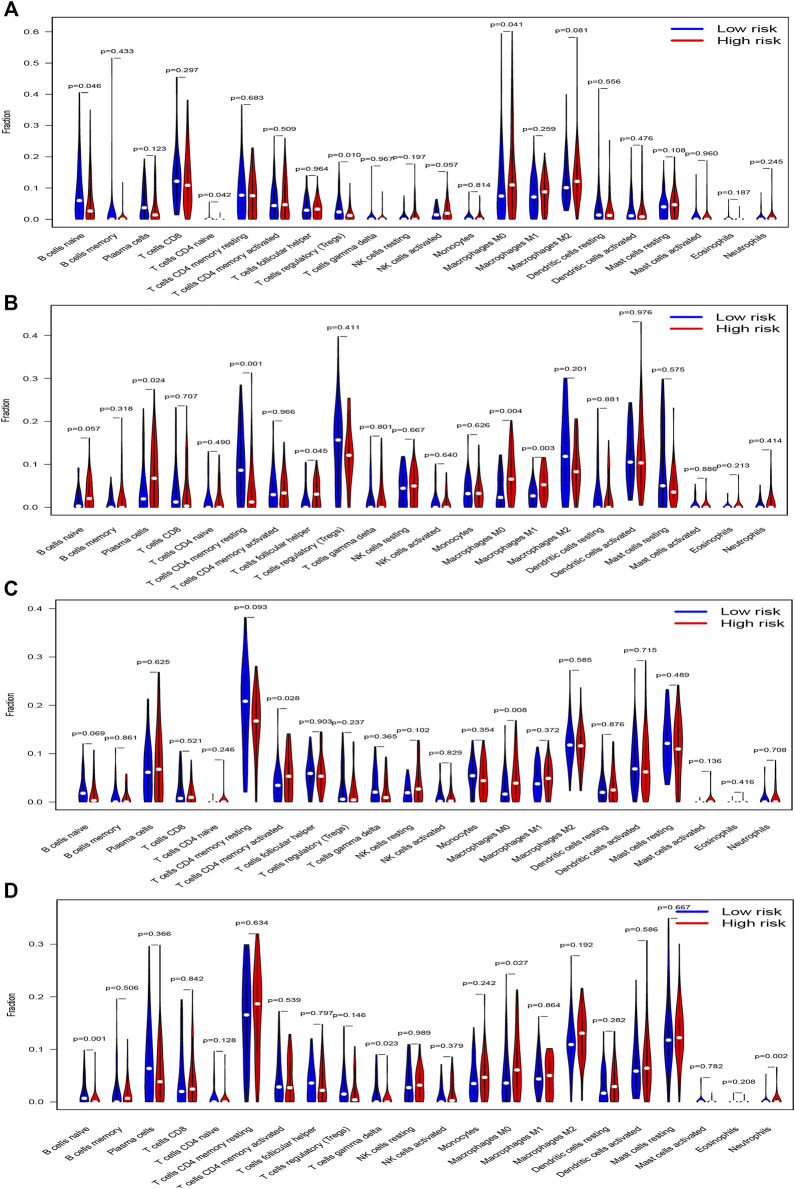
The vioplots of 22 immune cells contents of the high-risk and low-risk group in **(A)** TCGA dataset, **(B)** GSE13507, **(C)** GSE32548, and **(D)** GSE48075. M0 macrophages were all significantly different in these four datasets and M0 macrophages had higher infiltration in the high-risk group.

We used CIBERSORTx to evaluate 22 immune cell types in the training set and three validation sets. Patients with low risk scores had a higher infiltrated content of naive B cells, naive CD4^+^ T cells, and regulatory T cells compared with those with high risk scores, while the contents of infiltrated M0 macrophages were significantly higher in the high risk groups in the training datasets (Figure 9A). There were also 5 immune cell types significantly different between both risk groups in GSE13507, which included plasma cells, resting memory CD4^+^ T cells, follicular helper T cells, M0 macrophages, and M1 macrophages (Figure 9B). Activated memory CD4^+^ T cells and M0 macrophages were significantly different between both groups in GSE32548 (Figure 9C), and B cells naïve, gamma delta T cells, M0 macrophages, and neutrophils significantly differently infiltrated in both risk groups in GSE48075 (Figure 9D). Collectively, only M0 macrophages were all significantly different in these four datasets, which were significantly enriched in the high risk group.

### Tumor Mutational Burden Analysis

**FIGURE 10:**
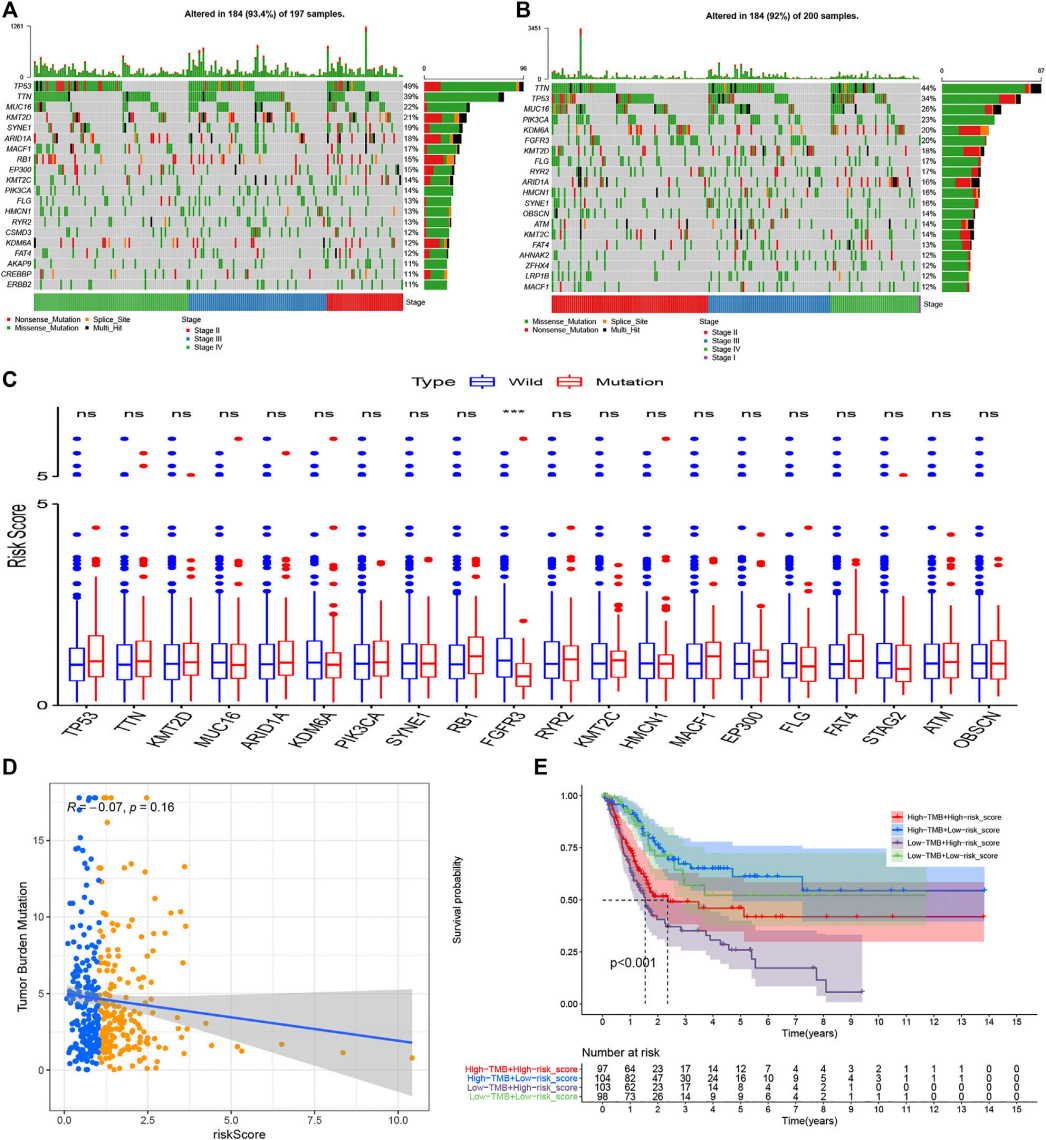
Tumor mutational burden analysis. The top 20 mutational genes were listed in the high risk group **(A)** and low risk group **(B)**. The risk scores between the wild type and the mutation type of the top frequent mutational genes were compared**(C)**. The risk scores were higher in the wild types of *FGFR3* compared with the mutational types of *FGFR3*. TMB decreased slightly with R = −0.07 and *p* = 0.16 **(D)**, and the Kaplan–Meier curves showed that patients with higher TMB and low-risk scores tended to have the best overall survival and those with low TMB and high risk scores usually had worse survival probabilities **(E)**. *p < 0.05; **p < 0.01; ***p < 0.001; ns: no significance.

The “Maftools” package in R software was used to analyze the mutational data in the TCGA dataset. Figures 10A,B show the top 20 mutational genes in the high and low risk groups respectively, and TP53, TTN, MUC16, KMT2D, SYNE1, ARID1A, MACF1, KMT2C, PIK3CA, FLG, HMCN1, RYR2, KDM6A, FAT4, were all top mutational genes and widely presented in both the high and low risk groups. In addition, RB1, EP300, CSMD3, AKAP9, ERBB2, and CREBBP belonged to the top 20 frequent mutational genes in the high risk group, while FGFR3, OBSCN, ATM, AHNAK2, ZFHX4, and LRP1B were part of the top 20 frequent mutational genes in the low risk group. Moreover, we also compared the risk scores between the wild- and mutational types of top mutational genes and found that the risk score in the EGFR-mutation type was significantly lower than that in the EGFR-wild type (Figure 10C). Furthermore, the scatter plot was used to explore the correlation between risk scores and TMB, and there was a slightly negative correlation between risk scores and TMB, although the correlation was not statistically significant (Figure 10D). Finally, the Kaplan–Meier curves showed that patients with higher TMB and low risk scores tended to have the best overall survival, and those with low TMB and high risk scores usually had a worse probability of survival (Figure 10E).

### Prediction of Immunotherapy Response

**FIGURE 11:**
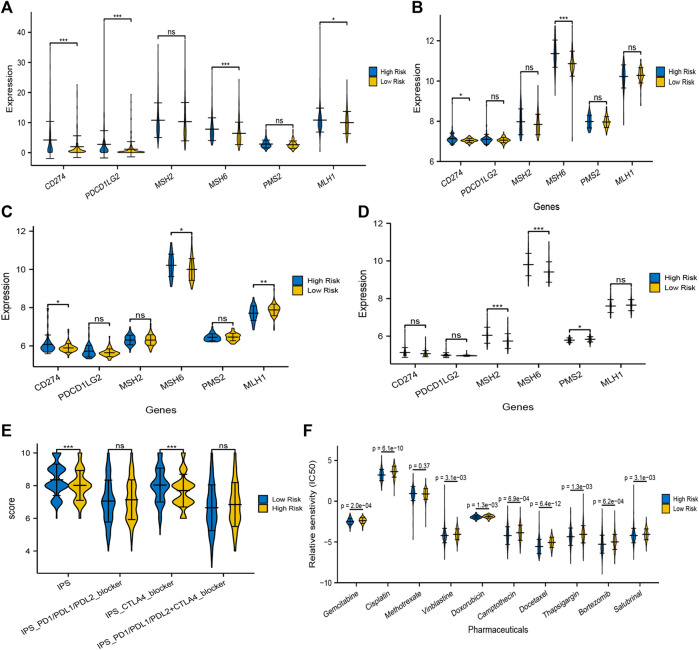
Prediction of immunotherapy and chemotherapy response. The expressions of PD-L1 and/or PD-L2 were significantly higher in the high risk group compared with that in the low risk group in training and validation sets **(A–D)**. At least one of the four mismatch repair genes, MLH1, MSH2, MSH6, and PMS2, in tumor samples expressed significantly higher in the high-risk group, which signified that microsatellites might be more stable in the high risk group. **(E)** The overall IPS and IPS for CTLA-4 blocker in the high risk group was significantly lower than that in the low risk group, predicting that patients with higher risk scores had a worse immunotherapy response. **(F)** Moreover, IC50 of gemcitabine, cisplatin, vinblastine, doxorubicin, camptothecin, docetaxel, bortezomib, thapsigargin, and salubrinal in the high-risk group were significantly lower than that in the low risk group, which meant that application of these drugs could be more beneficial to patients with higher risk scores.

Apart from TMB, Programmed cell death ligand 1 and ligand 2 (PD-L1 and PD-L2) and microsatellite instable (MSI) associated mismatch repair genes in tumor samples were identified as significant biomarkers to predict patients’ responses to immunotherapy. We then compared the transcriptional data of CD274 (PD-L1), PDCD1LG2 (PD-L2), MSH2, MSH6, PMS2, and MLH1 in the high and low risk groups. Interestingly, PD-L1 and PD-L2 in the high risk group were higher than that in the low risk group, while the expression of mismatch repair genes, MSH6 and MLH1, were significantly higher in the high risk group compared with that in the low risk group, which signified that the microsatellites might be more stable in the high-risk group (Figure 11A). Similarly, the expression of CD274 (PD-L1) was still higher in the high risk groups in GSE13507 and GSE32548, and one or more mismatch repair genes had higher expression in the high risk groups in GSE13507, GSE32548, and GSE48075 (Figures 11B–D).

TCIA, a database providing comprehensive immunogenomic analyses based on the TCGA, was employed to evaluate the immunotherapy response of patients with different risk scores by IPS. The total IPS and IPS for CTLA-4 blocker in the high-risk group was significantly lower than that in the low-risk group (Figure 11E), which powerfully predicted that patients with higher risk scores had a worse immunotherapy response, especially for CTLA-4 blockers. While there was no significant difference in the IPS for PD1/PD-L1/PD-L2 blocker and PD1/PD-L1/PD-L2 plus CTLA-4 blocker between both risk groups (Figure 11E). Taken together, TMB, MSI associated mismatch repair genes, and IPS all predicted that patients in the high risk score group might have a poor immunotherapy response, although the expression of PD-L1/PL-L2 was higher in tumor samples with high risk scores.

### Prediction of Chemotherapy Response

The “pRRophetic” package in R software was used to analyze the therapeutic biomarkers in the GDSC database and to investigate whether the UPR-related risk scores could predict the chemotherapy response of patients with different risk scores in the TCGA dataset. We first selected gemcitabine (G), cisplatin (C), methotrexate (M), vinblastine (V), and doxorubicin (A) to evaluate chemotherapy response in both risk groups, for they constituted the first-line chemotherapy regimen (GC or MVAC) in muscle-invasive bladder cancer. Moreover, camptothecin, docetaxel, bortezomib, thapsigargin, and salubrinal were also screened out to evaluate response, for bortezomib, thapsigargin, and salubrinal could induce UPR-related endoplasmic reticulum stress, and camptothecin and docetaxel were widely used in NMIBC. It is remarkable that the IC50 of almost all chemotherapy drugs mentioned above in the high risk group was significantly lower than that in the low risk group, which indicated that the application of these drugs could be more beneficial to patients with higher risk scores (Figure 11F).

### Predictive Nomogram Construction

**FIGURE 12:**
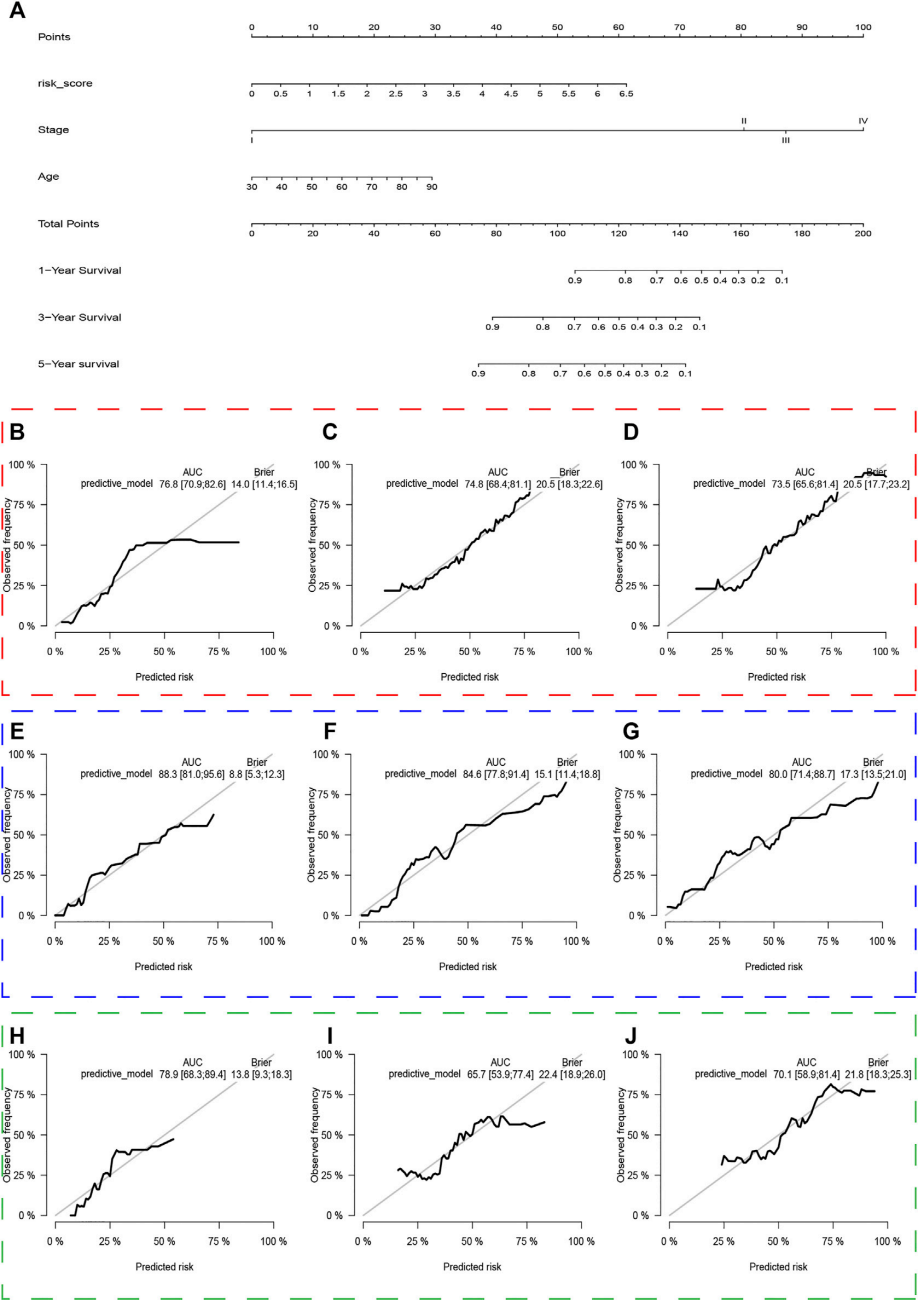
The nomogram of the UPR-related gene signature and its performance in the training set, and external validation sets. The predictive model was presented with a nomogram **(A)**. The red, blue and green dashed boxes corresponded to the TCGA dataset, GSE13507, and GSE48075. The AUC and Brier scores of the nomogram in years 1, 3, and 5 in the training set **(B–D)**, GSE13507 **(E–G)**, and GSE48075 **(H–J)**.

Age, AJCC-stage and the UPR-related risk score, as independent prognostic factors in the training set, were incorporated into the multivariate Cox regression model, which was presented in a nomogram to predict patients’ survival probabilities (Figure 12A). In the training set, the area under ROC (AUR) and Brier scores of the nomogram in 1, 3, 5 years were 76.8% [70.9%; 62.6%] and 14.0% [11.4%; 16.5%], 74.8% [68.4%; 81.1%] and 20.5% [18.3%; 22.6%], and 73.5% [65.6%; 81.4%] and 20.5% [17.7%; 23.2%], respectively (Figure12B–D). Subsequently, the enhanced bootstrap strategy was employed to validate the model internally. The optimism adjusted C statistics and the optimism adjusted Brier score in 1-, 3-, 5-years were 0.7648 and 0.1414, 0.7444 and 0.2081, 0.7306 and 0.1437 in internal validation, respectively (Table 2). Due to a lack of AJCC stage in GSE32548, only GSE13507 and GSE48075 were utilized as external validation sets to validate the nomogram. The AUR and Brier scores in 1-, 3-, 5-years were 88.3% [81.0%; 95.6%] and 8.8% [5.3%; 12.3%], 84.6% [77.8%; 91.4%] and 15.1% [11.4%; 18.8%], 80.0% [71.4%; 88.7%] and 17.3% [13.5%; 21.0%] in GSE13507 (Figure12E–G), which corresponded to 78.9% [68.3%; 89.4%] and 13.8% [9.3%; 18.3%], 65.7% [53.9%; 77.4%] and 22.4% [18.9%; 26.0%], 70.1% [58.9%; 81.4%] and 21.8% [18.3%; 25.3%] in GSE48075 (Figure12H–J).

**TABLE 2:** Basic parameters of enhanced bootstrap in internal validation.

Parameters	1-year	3-years	5-years
C harrell apparent	0.7178	0.7178	0.7178
C time apparent	0.7678	0.7476	0.7352
Brier apparent	0.1396	0.2047	0.1396
C harrell optimism	0.0005	−0.0001	0.0010
C time optimism	0.0030	0.0032	0.0046
Brier optimism	0.0019	−0.0034	−0.0042
Optimism adjusted overall C statistics	0.7173	0.7179	0.7168
Optimism adjusted C statistics at time	0.7648	0.7444	0.7306
Optimism adjusted brier score	0.1414	0.2081	0.1437

## Discussion

Bladder cancer is a common malignancy with high recurrence and invasiveness in the urinary system, with more than 80,500 new cases and 32,900 deaths in China each year (Chen et al., 2016; Siegel et al., 2017; Babjuk et al., 2019; Witjes et al., 2021). In recent years neoadjuvant chemotherapy, immunotherapy, and targeted therapy have been introduced into clinical practice, improving survival outcomes of BC patients to an extent, but the low response rate, non-negligible side effects, and heavy economic burden for patients prompted us to explore some useful biomarkers to screen for patients with good responses to immunotherapy or chemotherapy (Sievert et al., 2009; Pichler et al., 2018; Bednova and Leyton, 2020). To date, ERS associated UPR has attracted much attention from researchers and oncologists, as it could influence tumor proliferation and aggravation, and determine cell fate via three pathway branches initiated by IRE1α, PERK, and ATF6 (Urra et al., 2016; Oakes, 2020). A large number of UPR-related studies have been made in BC, including UPR-related biomarkers and drugs inducing ERS. Wang once reported that targeted inhibition of P4HB promoted cell sensitivity to gemcitabine and induced cell apoptosis via PERK/eIF2α/ATF4/CHOP signaling in urothelial carcinoma of the bladder (Wang et al., 2020), and Zhang discovered that OTUB1 facilitated bladder cancer progression by stabilizing ATF6 in response to endoplasmic reticulum stress (Zhang et al., 2021c). It has also been reported that some drugs have antitumor effects via activation of the ERS pathways, including nelfinavir-ritonavir combination (Sato et al., 2018), lopinavir-ritonavir combination (Okubo et al., 2019), Flaccidoxide-13-Acetate (Wu et al., 2019), Sulforaphane (Jo et al., 2014), and cantharidin (Su et al., 2015), etc.

Taking the significant roles of UPR into account, the present study constructed a UPR-related multigene signature to predict patients’ survival outcomes and the responses to immunotherapy and chemotherapy in the era of genomics and precision medicine. Herein, CEBPG, HYOU1, IMP3, KDELR3, MTHFD2, PDIA6, POP4, PREB, SRPRB, TATDN2, YIF1A, and ZBTB17 constituted a UPR signature with robust predictive performance. The ROC of the UPR signature in years 1, 3, 5 were 0.738, 0.648, and 0.667 respectively, which is much higher than that of classical clinicopathological parameters, such as age, grade, and AJCC-stage in the training set (Figure 7). Similarly, the good performance of the UPR signature was also validated in three validation sets, which illustrated the signature’s stability and wide applicability.

For a better understanding of the UPR-related signature, we investigated each gene involved in this signature. It was reported that CEBPG promoted esophageal squamous cell carcinoma progression, and the downregulation of CEBPG resulted in differentiation arrest in acute myeloid leukemia (Alberich-Jordà et al., 2012; Huang et al., 2020), although its function has still not been explored in BC. Asahi once discovered that the expression of HYOU1 was overexpressed and positively correlated with stage in BC (Asahi et al., 2002). IMP3 was widely studied and deemed as a biomarker with poor survival in both NMIBC and MIBC (Sitnikova et al., 2008; Szarvas et al., 2012; Yang et al., 2019). Zhang once revealed that KDELR3, as a risk gene, constituted a hypoxia signature that could robustly predict BC patients’ prognosis in the TCGA dataset (Zhang et al., 2021b). A Single nucleotide polymorphism study once revealed that increased risk in BC was associated with variants in MTHFD2 with OR = 1.7 (Andrew et al., 2009). Cheng once demonstrated that PDIA6 downregulation inhibited BC cell proliferation and invasion via the Wnt/β-catenin signaling pathway (Cheng et al., 2017). Meanwhile, Zhang discovered that lncRNA PCAT6 could regulate BC progression via the microRNA-143-3p/PDIA6 axis (Zhang et al., 2021d). Furthermore, POP4, PREB, SRPRB, TATDN2, YIF1A, and ZBTB17 were seldom studied in BC and perhaps researchers need to pay more attention to them in the future.

To investigate the relationship between the UPR-related signature and clinicopathological parameters, we first stratified patients with clinical parameters, and then the risk scores of patients in each subgroup were compared in the training and three validation sets. Interestingly, the risk scores in the subgroups of elder age, male, high grade, advanced stage, and metastasis were significantly higher than that in the other subgroups, which was also validated in the GEO datasets (Figure 4). Moreover, what attracted our attention was that the risk scores in the subgroups of basal type are significantly higher than that in luminal type, which indicates that our UPR-related risk score was closely related to the molecular subtypes used to predict treatment response and prognosis of BC patients (Robertson et al., 2017; Seiler et al., 2017; Kamoun et al., 2020). Secondly, we compared the survival outcomes of patients with higher or lower risk in the subgroups of age<70, age ≥ 70, female, male, high pathological grade, advanced stage, low T-stage, high T-stage, nodal metastasis-free, nodal metastasis, and metastasis-free, and found that patients in the high risk group still had a worse overall survival than ones in the low risk group (Figure 5), which demonstrated the stability and universality of the URP-related signature. In addition, the signature’s prognostic performance was also validated in the subgroups stratified by clinical parameters in GSE13507, GSE32548, and GSE48075 (Figure 6).

GSEA analysis was made with differentially expressed genes in the high- and low risk groups to seek out the enriched KEGG pathways in both groups. Immune, extracellular matrix, replication, and repair associated pathways were enriched into the high risk group in the training and validation sets, while metabolism related pathways belonged to the low risk group significantly. Extracellular matrix related pathways in the high risk groups included ECM receptor interaction, cell adhesion molecules, cytokine and cytokine receptor interaction, and so forth. These extracellular interactions led to direct or indirect control of tumor cellular activities such as adhesion, migration, metastasis, differentiation, proliferation, angiogenesis, and local immunosuppression in the tumor microenvironment (Lippitz, 2013; Hamidi and Ivaska, 2018; Läubli and Borsig, 2019), which uncovered the reason why patients with higher risk scores had worse survival to a certain extent. The replication and repair associated pathways enriched in the high risk group indicated that the tumors had high proliferation ability, strong mismatch repair ability, and stable microsatellite (Fishel, 2015; Li et al., 2016; Baretti and Le, 2018), which were in accordance with poor survival outcomes and poor immunotherapy response in the high risk group. Immune related pathways in the high risk groups included autoimmune disease pathways and inflammatory pathways, which had little correlation with immunosuppressive phenotype. As for the metabolism pathways enriched into the low risk groups, we believed that it revealed a status that high metabolic demand, oxidative stress, and disturbance of the protein-folding in the tumor microenvironment, which might induce ERS-related apoptosis in chronic or intense stress.

Immune cell infiltration in the tumor microenvironment plays a significant role in regulating the proliferation, invasion, and migration of cancer cells (Petrova et al., 2018; Liu et al., 2020). We compared the contents of 22 immune cells in both different risk groups and found that only M0 macrophage cells were significantly higher in the high risk groups in TCGA datasets and two validation datasets. Macrophages could be divided into 3 classes: M0, M1, and M2 macrophages (Locati et al., 2020). M0 macrophage was a kind of inactive macrophages that have the potential to differentiate M1 or M2 macrophages. M1 macrophages usually expressed MHC-II, CD68, CD80, and CD86 and had the effect of antitumor and pro-inflammation, while the M2 microphage in the tumor microenvironment could play a role in pro-tumor, immunosuppression, and anti-inflammation (Malfitano et al., 2020; Mantovani et al., 2021). In our study, M1 or M2 macrophages did not significantly enrich in the low or high risk groups respectively, but the contents of M0 macrophages were significantly higher in the high risk group, which indicates that M0 macrophages might correlate with poor prognosis. In most tumor microenvironments, tumor-associated macrophage (TAM) resembled M2-like macrophages and played a role in cancer progression, metastasis, and immunotherapy resistance (Liu et al., 2017). Li once reported that the low expression of M0 macrophage in tumor samples was associated with a better clinical prognosis in bladder cancer (Li et al., 2020). The two studies mentioned above agreed with our results.

TMB, PD-L1 and PD-L2, and MSI in tumor tissue are believed to be potent biomarkers for predicting immunotherapy response, but the relationship between these biomarkers is complex and it remains unclear whether employing a combination of biomarkers is superior to relying on a single marker (Luchini et al., 2019; Ren et al., 2020). In the present study, we first analyzed the difference of TMB in both risk groups. It was believed that tumors with more mutational genes tended to generate more mutational RNA and protein, which could be more easily recognized by the immune system and had a good response to immunotherapy (Rizvi et al., 2015). The top 20 mutational genes in both risk groups were listed to analyze the different mutational genes (Figures 10A,B), and then the risk scores between the wild types and the mutation types were compared. The risk scores in the wild type of FGFR3 were significantly higher than that in the mutation type of FGFR3 (Figure 10C). It has been reported that FGFR3 has a common mutational gene in bladder cancer and its mutation was associated with a favorable BC prognosis, which was in accordance with the low UPR-related risk score in the mutational type of EGFR3.

We also explored the correlation between UPR-related risk scores and TMB and found a negative correlation with R = −0.07. Subsequently, the transcriptome data of significant DNA mismatch repair genes (MLH1, MSH2, MSH6, and PMS2) were compared, and we found that one or more mismatch repair genes had higher expression in the high risk groups, which signified that microsatellites might be more stable in the high-risk group. After that, the immune-comprehensive analysis from the TCIA database also showed that overall IPS and IPS for CTLA4 blocker were significantly higher in the low risk group, which indicated that patients with lower risk scores had a better immunotherapy response. Interestingly, we discovered that the expression of PD-L1 and/or PD-L2 were significantly higher in the high risk group than that in the low risk group, and whether it meant that patients with higher risk scores had a better response to immunotherapy, at least anti-PD-1/PD-L immune checkpoint therapy (Figures 11A–D). This contradicted the TMB and MSI analysis, and IPS for PD-1/PD-L1/PD-L2 also showed no significant difference in both risk groups. Keenan once explored the genomic correlates of response to immune checkpoint blockade and found that as a predictive biomarker for PD-l/PD-L1 therapy, PD-L1 lacked diagnostic accuracy in selecting patients with good responses (Keenan et al., 2019). The predictive performance of PD-L1 seemed to depend on tumor type and specific immune checkpoint inhibitors (Chan et al., 2019). It was based on the fact mentioned above that MSI was regarded as a premier biomarker for predicting the response to immunotherapy in current clinical practice, irrespective of tumor origin (Duffy and Crown, 2019).

Prediction of chemotherapy response showed that nearly all first-line chemotherapeutic had a better response to patients with higher risk scores, which indicated that the UPR-related signature closely correlated with chemotherapy response. Finally, a predictive model for risk score, age, and stage was constructed in the training set and presented in a nomogram. The Enhanced bootstrap with 1,000 times was used to validate the nomogram internally, and GSE13507 and GSE48075 were utilized as external validation sets to validate the nomogram. Whatever the training dataset, the internal validation dataset, or the external validation dataset, the performance of this model is still steady and robust (Figure 12).

This is the first UPR-related signature in bladder cancer, which could accurately predict the survival outcomes of BC patients compared with the traditional pathological parameters. Moreover, the molecular signature has close relationships with clinicopathological parameters and closely correlates with the response to immunotherapy and chemotherapy.

## Data Availability

The original contributions presented in the study are included in the article/Supplementary Material, further inquiries can be directed to the corresponding authors.
