# Supplementary material for: An Unfolded Protein Response Related Signature Could Robustly Predict Survival Outcomes and Closely Correlate With Response to Immunotherapy and Chemotherapy in Bladder Cancer
Source: Front Mol Biosci. 2021 Dec 23;8:780329. doi: 10.3389/fmolb.2021.780329 (PMC8732996; doi:10.3389/fmolb.2021.780329)
Supplement: Supplementary file 1 [file DataSheet1.docx]

Supplementary Table 1. The Matrix Expression Screened by Lasso Regression

| ID | futime | fustat | CEBPG | HYOU1 | IMP3 | KDELR3 | MTHFD2 | PDIA6 | POP4 | PREB | SRPRB­ | TATDN2 | YIF1A | ZBTB17 |
| --- | --- | --- | --- | --- | --- | --- | --- | --- | --- | --- | --- | --- | --- | --- |
| TCGA-DK-AA76 | 366 | 0 | 13.601 | 13.216 | 11.112 | 8.861 | 10.807 | 12.880 | 11.120 | 11.137 | 11.623 | 11.747 | 10.689 | 10.770 |
| TCGA-FD-A3SJ | 739 | 1 | 11.536 | 13.420 | 12.267 | 9.212 | 12.481 | 13.945 | 11.404 | 12.982 | 12.501 | 11.739 | 12.576 | 12.284 |
| TCGA-E7-A3X6 | 407 | 0 | 11.369 | 14.049 | 11.504 | 8.543 | 11.161 | 13.845 | 10.670 | 11.543 | 12.179 | 10.926 | 11.727 | 11.141 |
| TCGA-E7-A97Q | 246 | 1 | 11.345 | 12.449 | 10.611 | 11.214 | 11.041 | 12.812 | 10.027 | 11.948 | 11.027 | 11.788 | 12.087 | 10.826 |
| TCGA-KQ-A41S | 35 | 0 | 11.436 | 12.977 | 11.089 | 11.177 | 11.293 | 13.716 | 10.066 | 11.429 | 11.726 | 10.709 | 11.829 | 10.902 |
| TCGA-XF-A9SJ | 98 | 1 | 11.246 | 12.693 | 10.617 | 11.126 | 11.774 | 13.836 | 10.883 | 12.199 | 12.049 | 10.825 | 12.393 | 10.524 |
| TCGA-FD-A62O | 216 | 1 | 10.698 | 12.319 | 10.786 | 10.903 | 11.273 | 13.403 | 9.767 | 11.075 | 11.380 | 10.297 | 11.024 | 10.031 |
| TCGA-GU-A42P | 332 | 1 | 12.315 | 13.633 | 11.599 | 11.597 | 11.176 | 13.860 | 10.744 | 12.365 | 12.166 | 11.951 | 12.237 | 11.778 |
| TCGA-FD-A3B3 | 392 | 0 | 12.172 | 14.541 | 11.405 | 9.976 | 12.487 | 14.200 | 12.615 | 12.137 | 12.918 | 11.120 | 12.767 | 11.443 |
| TCGA-XF-A9T2 | 575 | 1 | 10.260 | 12.296 | 10.013 | 8.308 | 11.291 | 12.363 | 9.055 | 12.979 | 10.410 | 9.973 | 10.786 | 9.760 |
| TCGA-FJ-A3Z7 | 945 | 0 | 12.435 | 12.246 | 10.808 | 10.844 | 12.254 | 11.320 | 10.263 | 11.466 | 10.962 | 12.510 | 11.154 | 11.217 |
| TCGA-CF-A9FL | 565 | 1 | 11.099 | 12.461 | 11.288 | 9.671 | 10.119 | 12.859 | 10.090 | 11.266 | 11.100 | 9.884 | 11.242 | 10.243 |
| TCGA-C4-A0F7 | 62 | 1 | 11.977 | 13.548 | 11.198 | 8.093 | 12.296 | 14.480 | 10.786 | 12.728 | 13.627 | 11.805 | 11.464 | 10.274 |
| TCGA-E7-A8O8 | 13 | 0 | 11.444 | 11.687 | 11.171 | 8.150 | 9.790 | 11.097 | 10.461 | 11.086 | 11.631 | 11.361 | 11.634 | 10.313 |
| TCGA-XF-A9SY | 640 | 0 | 11.611 | 14.144 | 10.330 | 9.602 | 12.411 | 14.295 | 11.293 | 11.495 | 11.428 | 10.167 | 11.819 | 10.211 |
| TCGA-FD-A5BU | 476 | 0 | 11.417 | 12.484 | 11.636 | 9.470 | 11.280 | 13.157 | 10.261 | 11.528 | 11.426 | 10.521 | 11.864 | 10.007 |
| TCGA-ZF-A9R9 | 864 | 1 | 10.295 | 11.155 | 9.911 | 8.811 | 9.834 | 11.353 | 9.572 | 10.974 | 10.274 | 9.728 | 10.681 | 10.162 |
| TCGA-GV-A3QH | 258 | 1 | 12.835 | 13.693 | 11.394 | 12.380 | 12.440 | 14.465 | 11.622 | 12.884 | 12.896 | 11.571 | 12.765 | 11.637 |
| TCGA-UY-A78L | 1127 | 0 | 12.166 | 13.374 | 11.116 | 11.225 | 11.543 | 13.572 | 11.470 | 12.042 | 12.608 | 11.387 | 12.727 | 10.974 |
| TCGA-4Z-AA7R | 522 | 1 | 11.778 | 14.226 | 11.466 | 10.873 | 11.890 | 14.426 | 10.689 | 11.823 | 11.715 | 11.197 | 11.502 | 11.362 |
| TCGA-FD-A3B5 | 272 | 1 | 12.102 | 13.452 | 11.653 | 10.710 | 12.166 | 14.745 | 10.715 | 12.301 | 11.940 | 11.513 | 12.396 | 10.595 |
| TCGA-PQ-A6FI | 372 | 0 | 11.608 | 12.877 | 12.115 | 8.219 | 12.692 | 14.376 | 11.069 | 12.277 | 12.872 | 11.450 | 12.599 | 10.698 |
| TCGA-XF-A8HI | 544 | 1 | 11.211 | 12.838 | 11.341 | 7.500 | 9.000 | 12.872 | 10.078 | 11.288 | 11.206 | 10.311 | 11.057 | 10.644 |
| TCGA-BL-A0C8 | 876 | 0 | 11.338 | 12.968 | 9.337 | 10.162 | 10.444 | 12.051 | 9.636 | 10.047 | 10.726 | 9.677 | 9.746 | 9.208 |
| TCGA-DK-AA6U | 578 | 0 | 12.235 | 14.164 | 11.668 | 11.763 | 11.715 | 13.819 | 14.819 | 12.268 | 12.138 | 11.559 | 11.923 | 11.118 |
| TCGA-BL-A13I | 223 | 1 | 9.490 | 11.521 | 8.846 | 8.971 | 10.457 | 11.693 | 8.792 | 9.838 | 10.481 | 9.948 | 9.372 | 8.601 |
| TCGA-GC-A3I6 | 630 | 1 | 11.670 | 13.056 | 11.159 | 9.488 | 11.587 | 12.839 | 10.539 | 11.328 | 11.697 | 10.202 | 11.171 | 10.157 |
| TCGA-H4-A2HO | 46 | 0 | 10.866 | 12.345 | 10.830 | 9.917 | 9.510 | 11.929 | 10.122 | 10.853 | 11.506 | 10.247 | 10.988 | 10.498 |
| TCGA-UY-A78O | 2312 | 0 | 11.312 | 12.167 | 11.120 | 8.500 | 10.102 | 12.182 | 10.696 | 11.144 | 11.195 | 10.849 | 11.817 | 10.297 |
| TCGA-BT-A3PH | 142 | 1 | 12.267 | 13.313 | 11.854 | 11.393 | 12.515 | 13.244 | 10.928 | 12.606 | 13.129 | 11.192 | 14.280 | 11.091 |
| TCGA-FD-A3SN | 390 | 0 | 11.826 | 13.723 | 10.561 | 9.522 | 12.377 | 13.608 | 11.183 | 12.169 | 12.518 | 11.263 | 12.276 | 11.027 |
| TCGA-XF-AAN5 | 2293 | 0 | 10.534 | 11.360 | 10.276 | 7.748 | 11.168 | 11.742 | 9.382 | 10.257 | 10.197 | 9.711 | 9.890 | 8.655 |
| TCGA-XF-AAN3 | 2625 | 0 | 12.398 | 14.225 | 12.277 | 10.569 | 12.361 | 14.695 | 11.989 | 13.104 | 13.264 | 11.009 | 12.601 | 12.152 |
| TCGA-ZF-AA4W | 1830 | 0 | 11.478 | 12.586 | 11.845 | 8.611 | 11.494 | 12.394 | 10.713 | 11.867 | 12.629 | 9.512 | 11.464 | 10.709 |
| TCGA-E7-A6MD | 129 | 0 | 11.891 | 12.345 | 11.691 | 10.364 | 11.154 | 12.774 | 10.920 | 11.609 | 12.061 | 11.413 | 11.809 | 11.124 |
| TCGA-KQ-A41O | 1538 | 0 | 11.695 | 10.919 | 11.533 | 10.996 | 10.610 | 11.199 | 10.523 | 11.687 | 11.546 | 11.311 | 11.826 | 10.830 |
| TCGA-E7-A5KF | 20 | 0 | 11.647 | 12.096 | 11.972 | 7.788 | 9.058 | 13.400 | 10.576 | 11.305 | 11.048 | 10.647 | 11.300 | 10.634 |
| TCGA-YF-AA3M | 415 | 0 | 10.889 | 13.430 | 9.838 | 8.308 | 11.348 | 12.966 | 10.063 | 10.603 | 11.692 | 10.040 | 11.547 | 10.554 |
| TCGA-CF-A3MF | 383 | 0 | 10.047 | 9.132 | 9.535 | 6.977 | 6.728 | 7.672 | 9.576 | 10.102 | 10.085 | 10.062 | 9.615 | 10.472 |
| TCGA-DK-AA6R | 5041 | 0 | 11.962 | 12.482 | 10.515 | 10.107 | 12.030 | 13.233 | 10.893 | 11.506 | 11.983 | 10.504 | 11.661 | 10.142 |
| TCGA-FD-A3SR | 602 | 1 | 12.704 | 14.076 | 11.619 | 10.948 | 11.814 | 14.365 | 11.851 | 12.392 | 11.950 | 10.875 | 12.041 | 11.194 |
| TCGA-DK-A3X2 | 547 | 1 | 11.688 | 13.381 | 11.520 | 8.267 | 13.660 | 13.948 | 10.622 | 12.410 | 10.862 | 11.409 | 12.192 | 11.247 |
| TCGA-YC-A8S6 | 293 | 0 | 11.348 | 12.401 | 10.412 | 8.304 | 10.590 | 12.236 | 9.773 | 10.729 | 11.038 | 10.217 | 10.914 | 10.647 |
| TCGA-GV-A3JV | 434 | 1 | 12.504 | 12.553 | 11.280 | 10.137 | 10.934 | 12.328 | 11.088 | 11.254 | 12.198 | 11.635 | 12.064 | 10.775 |
| TCGA-4Z-AA84 | 460 | 0 | 12.788 | 12.742 | 10.764 | 11.335 | 12.694 | 12.805 | 11.038 | 11.623 | 11.814 | 10.920 | 11.877 | 10.128 |
| TCGA-FD-A5BV | 163 | 1 | 11.109 | 11.831 | 10.638 | 9.653 | 10.832 | 12.093 | 9.574 | 10.841 | 11.517 | 10.696 | 11.557 | 9.858 |
| TCGA-4Z-AA7W | 840 | 0 | 11.243 | 12.072 | 11.581 | 9.892 | 11.283 | 12.471 | 10.547 | 12.355 | 12.312 | 11.425 | 12.889 | 10.341 |
| TCGA-ZF-AA4U | 262 | 1 | 11.725 | 12.777 | 11.588 | 9.006 | 10.435 | 13.459 | 10.508 | 11.661 | 11.762 | 10.378 | 11.265 | 10.437 |
| TCGA-KQ-A41R | 1350 | 0 | 10.815 | 10.619 | 10.639 | 8.607 | 9.831 | 10.474 | 9.982 | 11.569 | 10.601 | 10.816 | 10.311 | 10.364 |
| TCGA-GV-A3JZ | 603 | 0 | 12.908 | 14.158 | 11.543 | 12.191 | 12.688 | 14.227 | 13.545 | 11.984 | 12.017 | 11.634 | 12.596 | 11.308 |
| TCGA-GD-A3OQ | 95 | 0 | 12.076 | 13.954 | 11.730 | 9.957 | 11.523 | 13.970 | 10.784 | 11.854 | 12.006 | 10.923 | 11.859 | 10.962 |
| TCGA-ZF-A9R4 | 921 | 0 | 11.390 | 13.177 | 11.122 | 9.524 | 11.001 | 13.220 | 10.694 | 11.549 | 12.293 | 11.760 | 11.457 | 11.157 |
| TCGA-FD-A6TG | 93 | 1 | 11.964 | 14.423 | 11.565 | 9.674 | 11.617 | 14.176 | 11.152 | 12.240 | 12.272 | 10.877 | 12.055 | 10.965 |
| TCGA-CU-A72E | 413 | 1 | 11.495 | 12.870 | 11.775 | 9.886 | 10.819 | 13.228 | 10.885 | 11.972 | 12.455 | 10.716 | 11.993 | 10.927 |
| TCGA-UY-A78M | 690 | 1 | 12.465 | 13.091 | 12.248 | 11.699 | 11.665 | 13.442 | 10.741 | 12.115 | 11.675 | 10.933 | 12.817 | 11.156 |
| TCGA-E7-A7XN | 428 | 0 | 11.730 | 12.862 | 11.271 | 6.845 | 12.544 | 13.357 | 10.741 | 11.787 | 11.750 | 10.930 | 11.811 | 10.583 |
| TCGA-XF-AAMX | 206 | 1 | 11.754 | 15.242 | 9.699 | 10.228 | 8.910 | 13.403 | 10.925 | 11.992 | 11.853 | 11.302 | 11.945 | 11.523 |
| TCGA-FD-A43S | 455 | 0 | 10.776 | 12.229 | 11.986 | 9.281 | 9.980 | 12.389 | 10.814 | 11.832 | 11.855 | 10.994 | 12.165 | 11.042 |
| TCGA-FD-A43N | 699 | 0 | 11.792 | 13.472 | 11.195 | 11.523 | 11.335 | 13.508 | 10.764 | 11.842 | 11.417 | 11.039 | 12.287 | 11.583 |
| TCGA-FT-A61P | 337 | 0 | 11.065 | 12.841 | 10.499 | 11.159 | 11.918 | 13.824 | 10.447 | 11.955 | 11.241 | 10.850 | 12.137 | 10.391 |
| TCGA-ZF-AA58 | 1649 | 0 | 12.023 | 13.052 | 10.536 | 10.338 | 12.822 | 13.155 | 11.336 | 11.001 | 12.400 | 11.157 | 12.323 | 10.970 |
| TCGA-FD-A6TE | 376 | 0 | 12.013 | 13.973 | 11.752 | 8.308 | 11.849 | 14.391 | 11.140 | 12.367 | 11.976 | 11.639 | 11.894 | 11.229 |
| TCGA-GV-A3JW | 649 | 0 | 12.377 | 13.448 | 12.142 | 10.635 | 11.752 | 14.993 | 12.732 | 11.845 | 12.608 | 13.095 | 12.167 | 10.992 |
| TCGA-BT-A20T | 453 | 1 | 10.773 | 11.347 | 11.378 | 9.351 | 9.910 | 12.032 | 9.692 | 10.964 | 10.914 | 10.269 | 10.757 | 9.895 |
| TCGA-DK-AA75 | 340 | 1 | 11.602 | 11.738 | 10.507 | 8.833 | 10.674 | 11.450 | 10.445 | 11.422 | 10.304 | 9.362 | 11.947 | 11.176 |
| TCGA-BT-A20X | 251 | 1 | 12.496 | 13.227 | 11.267 | 9.288 | 12.044 | 13.061 | 11.283 | 11.331 | 12.739 | 10.416 | 12.404 | 10.613 |
| TCGA-FD-A6TI | 294 | 1 | 11.337 | 13.807 | 11.849 | 8.527 | 10.743 | 13.449 | 10.969 | 11.975 | 12.377 | 10.959 | 12.696 | 10.773 |
| TCGA-BT-A0S7 | 200 | 1 | 10.383 | 12.770 | 9.794 | 10.093 | 11.602 | 13.629 | 9.250 | 10.710 | 11.761 | 9.864 | 11.323 | 8.229 |
| TCGA-DK-AA6P | 457 | 0 | 12.783 | 12.383 | 11.510 | 10.943 | 11.202 | 13.040 | 10.675 | 11.226 | 11.521 | 10.641 | 9.992 | 10.805 |
| TCGA-XF-AAME | 2828 | 1 | 11.321 | 13.621 | 10.934 | 11.331 | 12.065 | 14.063 | 10.633 | 11.985 | 12.468 | 10.703 | 11.920 | 10.483 |
| TCGA-DK-A1AC | 3981 | 0 | 12.590 | 13.493 | 11.881 | 10.401 | 13.743 | 14.209 | 11.158 | 12.696 | 12.530 | 11.148 | 12.425 | 11.975 |
| TCGA-XF-AAMJ | 1670 | 1 | 10.301 | 12.261 | 10.553 | 10.896 | 10.474 | 12.498 | 9.526 | 11.030 | 11.404 | 10.211 | 11.351 | 10.065 |
| TCGA-XF-A9SU | 182 | 1 | 11.147 | 12.099 | 10.851 | 9.801 | 10.482 | 12.032 | 10.596 | 11.464 | 11.983 | 10.686 | 12.038 | 10.679 |
| TCGA-ZF-A9RF | 1949 | 0 | 11.736 | 13.167 | 11.112 | 9.585 | 11.527 | 14.185 | 12.708 | 11.708 | 12.543 | 11.214 | 12.140 | 10.920 |
| TCGA-4Z-AA7M | 405 | 0 | 11.422 | 13.002 | 10.560 | 9.313 | 10.668 | 13.291 | 12.831 | 11.425 | 11.583 | 11.692 | 11.024 | 10.852 |
| TCGA-BT-A2LA | 522 | 0 | 13.836 | 13.717 | 11.687 | 9.447 | 12.663 | 13.714 | 12.677 | 12.514 | 12.394 | 12.145 | 12.374 | 12.252 |
| TCGA-GC-A3RC | 484 | 0 | 12.321 | 13.947 | 10.733 | 10.062 | 11.952 | 13.218 | 10.570 | 11.177 | 11.326 | 11.005 | 12.051 | 10.812 |
| TCGA-CF-A47X | 384 | 0 | 12.649 | 12.617 | 12.151 | 5.807 | 11.563 | 13.029 | 10.880 | 12.208 | 11.560 | 11.415 | 11.554 | 10.850 |
| TCGA-DK-A3IU | 609 | 0 | 11.123 | 13.127 | 10.992 | 11.926 | 12.131 | 13.510 | 10.743 | 11.626 | 12.054 | 11.018 | 12.369 | 11.114 |
| TCGA-E5-A4U1 | 1181 | 0 | 11.592 | 10.499 | 11.872 | 7.476 | 10.147 | 10.765 | 10.664 | 11.964 | 11.456 | 10.751 | 11.948 | 11.874 |
| TCGA-5N-A9KM | 530 | 1 | 11.023 | 12.403 | 11.470 | 11.510 | 11.159 | 13.075 | 11.623 | 11.648 | 12.166 | 10.706 | 11.397 | 10.218 |
| TCGA-DK-A6B5 | 1249 | 0 | 12.617 | 14.340 | 11.116 | 11.162 | 11.390 | 14.825 | 11.240 | 12.533 | 12.705 | 12.991 | 12.237 | 11.517 |
| TCGA-BT-A42E | 738 | 0 | 12.601 | 11.297 | 11.078 | 10.489 | 12.634 | 12.657 | 11.220 | 11.465 | 12.353 | 10.949 | 13.010 | 10.505 |
| TCGA-ZF-AA4T | 599 | 1 | 12.241 | 12.289 | 11.970 | 9.695 | 11.575 | 13.234 | 11.006 | 12.567 | 11.700 | 11.953 | 11.059 | 11.083 |
| TCGA-G2-AA3B | 2008 | 0 | 12.590 | 13.067 | 11.979 | 12.703 | 11.634 | 12.614 | 11.665 | 12.081 | 12.092 | 10.726 | 12.846 | 11.120 |
| TCGA-DK-A3IN | 250 | 1 | 11.324 | 13.112 | 11.159 | 11.105 | 12.059 | 13.716 | 10.548 | 12.496 | 12.052 | 12.681 | 12.077 | 11.297 |
| TCGA-G2-A2EJ | 931 | 0 | 12.276 | 13.681 | 11.976 | 8.842 | 12.595 | 13.971 | 11.129 | 11.876 | 12.995 | 10.525 | 12.077 | 11.219 |
| TCGA-GD-A76B | 224 | 0 | 12.180 | 13.202 | 11.953 | 10.022 | 10.435 | 12.915 | 11.694 | 11.849 | 12.226 | 11.567 | 12.091 | 10.222 |
| TCGA-GC-A3RB | 582 | 0 | 12.176 | 14.681 | 11.928 | 11.563 | 14.031 | 14.988 | 13.322 | 12.536 | 12.790 | 12.486 | 12.042 | 11.195 |
| TCGA-LT-A5Z6 | 474 | 0 | 12.267 | 12.844 | 11.150 | 12.243 | 11.327 | 13.082 | 10.852 | 11.831 | 11.284 | 10.636 | 11.329 | 10.614 |
| TCGA-FD-A3SS | 391 | 1 | 12.620 | 13.702 | 11.730 | 11.991 | 12.808 | 14.719 | 11.289 | 12.879 | 12.270 | 11.591 | 12.738 | 11.208 |
| TCGA-FD-A5BY | 251 | 0 | 11.854 | 12.546 | 10.803 | 9.098 | 11.707 | 13.707 | 11.892 | 12.135 | 12.373 | 10.842 | 11.854 | 11.097 |
| TCGA-FD-A43Y | 369 | 0 | 11.749 | 12.784 | 11.065 | 9.812 | 11.407 | 13.383 | 10.431 | 11.043 | 11.360 | 11.561 | 12.145 | 10.171 |
| TCGA-GC-A3BM | 651 | 1 | 11.622 | 12.883 | 11.229 | 8.974 | 10.992 | 13.577 | 10.804 | 11.697 | 11.860 | 10.815 | 11.907 | 10.457 |
| TCGA-DK-A1A6 | 2020 | 0 | 13.245 | 13.525 | 11.744 | 10.054 | 12.785 | 13.840 | 11.528 | 12.369 | 12.536 | 11.772 | 11.420 | 10.226 |
| TCGA-BT-A42F | 864 | 0 | 12.187 | 12.920 | 13.075 | 9.441 | 13.196 | 13.444 | 11.148 | 12.428 | 12.630 | 11.025 | 13.080 | 10.924 |
| TCGA-DK-A1AB | 508 | 1 | 12.361 | 13.699 | 11.796 | 11.278 | 12.264 | 14.284 | 11.329 | 12.390 | 12.690 | 10.467 | 11.116 | 10.953 |
| TCGA-GD-A3OS | 638 | 0 | 12.103 | 13.848 | 11.346 | 12.240 | 12.883 | 14.473 | 10.855 | 12.262 | 12.782 | 11.753 | 12.303 | 11.642 |
| TCGA-G2-A2EL | 819 | 1 | 12.879 | 14.604 | 10.459 | 6.700 | 13.866 | 12.845 | 11.376 | 11.930 | 12.079 | 11.348 | 12.161 | 12.229 |
| TCGA-GD-A2C5 | 812 | 0 | 11.778 | 13.378 | 12.151 | 11.435 | 11.575 | 13.932 | 11.303 | 12.252 | 12.451 | 10.508 | 12.494 | 11.304 |
| TCGA-FD-A5BX | 173 | 1 | 10.371 | 12.904 | 10.899 | 10.531 | 11.603 | 13.188 | 10.200 | 11.410 | 12.250 | 10.631 | 12.424 | 10.925 |
| TCGA-E7-A6MF | 436 | 0 | 11.177 | 12.521 | 11.208 | 6.895 | 8.044 | 12.681 | 10.230 | 10.886 | 11.136 | 10.769 | 10.497 | 10.414 |
| TCGA-K4-A3WS | 761 | 0 | 12.218 | 13.233 | 10.389 | 10.746 | 11.450 | 12.659 | 10.808 | 11.825 | 11.547 | 10.800 | 11.659 | 10.920 |
| TCGA-GC-A3OO | 481 | 0 | 11.117 | 13.171 | 10.610 | 10.577 | 10.838 | 13.050 | 10.216 | 10.935 | 12.051 | 10.888 | 11.507 | 10.596 |
| TCGA-BT-A20V | 154 | 1 | 11.771 | 13.555 | 10.542 | 8.626 | 12.632 | 14.259 | 11.080 | 11.881 | 11.842 | 9.522 | 9.899 | 9.814 |
| TCGA-2F-A9KR | 3148 | 0 | 11.686 | 12.251 | 11.675 | 8.974 | 9.209 | 12.966 | 10.473 | 11.759 | 11.911 | 11.293 | 10.678 | 10.709 |
| TCGA-CF-A1HS | 382 | 0 | 11.945 | 12.183 | 10.500 | 9.825 | 12.241 | 13.325 | 10.577 | 11.537 | 12.065 | 10.109 | 11.111 | 9.788 |
| TCGA-CF-A3MG | 369 | 0 | 11.706 | 12.513 | 11.328 | 7.200 | 10.285 | 13.273 | 10.873 | 11.609 | 11.265 | 10.579 | 10.781 | 10.466 |
| TCGA-XF-AAN4 | 823 | 1 | 10.833 | 12.034 | 9.867 | 9.457 | 11.419 | 12.396 | 9.714 | 10.868 | 10.987 | 10.021 | 10.617 | 9.812 |
| TCGA-UY-A9PH | 1561 | 0 | 11.476 | 11.963 | 10.982 | 8.331 | 10.001 | 11.731 | 10.214 | 10.330 | 11.349 | 10.170 | 11.194 | 10.105 |
| TCGA-K4-A5RH | 276 | 0 | 12.602 | 14.451 | 11.254 | 11.626 | 13.631 | 14.614 | 11.893 | 12.357 | 12.991 | 12.926 | 12.937 | 12.530 |
| TCGA-CU-A5W6 | 56 | 1 | 11.932 | 13.449 | 12.090 | 10.399 | 11.299 | 13.332 | 11.339 | 12.005 | 12.333 | 10.690 | 12.274 | 10.540 |
| TCGA-E7-A678 | 425 | 0 | 10.230 | 11.503 | 11.637 | 6.524 | 7.852 | 11.736 | 10.247 | 10.796 | 11.142 | 10.362 | 11.487 | 10.051 |
| TCGA-DK-A1A3 | 665 | 1 | 11.316 | 13.861 | 11.241 | 12.849 | 11.791 | 13.632 | 12.011 | 12.130 | 12.499 | 9.326 | 12.375 | 9.890 |
| TCGA-GU-AATO | 324 | 1 | 10.198 | 12.439 | 10.240 | 8.558 | 10.380 | 13.733 | 10.283 | 12.534 | 11.798 | 10.502 | 11.311 | 10.410 |
| TCGA-4Z-AA7N | 1367 | 1 | 10.410 | 12.087 | 9.753 | 9.541 | 10.263 | 11.740 | 9.093 | 10.549 | 10.191 | 10.095 | 10.249 | 9.812 |
| TCGA-XF-AAN0 | 1718 | 1 | 11.838 | 13.037 | 11.363 | 11.840 | 11.364 | 13.625 | 11.390 | 12.287 | 11.898 | 10.718 | 12.331 | 10.807 |
| TCGA-XF-AAMZ | 1348 | 1 | 11.452 | 12.250 | 10.785 | 8.257 | 10.889 | 12.268 | 10.397 | 10.818 | 10.815 | 9.651 | 10.577 | 9.984 |
| TCGA-GU-A762 | 232 | 1 | 11.211 | 14.121 | 11.473 | 9.591 | 11.983 | 14.008 | 10.969 | 12.066 | 11.946 | 11.401 | 13.160 | 10.976 |
| TCGA-K4-A4AB | 76 | 0 | 11.222 | 12.583 | 10.753 | 9.384 | 9.794 | 13.462 | 10.940 | 11.413 | 10.936 | 10.465 | 11.756 | 10.681 |
| TCGA-BT-A20N | 795 | 1 | 12.082 | 13.099 | 10.656 | 9.923 | 12.189 | 14.072 | 11.033 | 11.310 | 12.393 | 11.066 | 11.625 | 10.577 |
| TCGA-FD-A6TC | 187 | 0 | 11.312 | 13.092 | 11.063 | 10.331 | 12.047 | 13.803 | 10.937 | 12.699 | 12.636 | 10.380 | 11.605 | 11.329 |
| TCGA-4Z-AA7O | 399 | 0 | 10.507 | 11.605 | 10.699 | 8.697 | 10.004 | 12.483 | 9.656 | 10.893 | 10.946 | 9.437 | 10.736 | 9.664 |
| TCGA-XF-A8HD | 2964 | 0 | 11.240 | 13.283 | 10.464 | 11.769 | 12.291 | 13.839 | 10.992 | 12.201 | 12.154 | 10.729 | 12.448 | 11.202 |
| TCGA-DK-A3IL | 413 | 1 | 11.468 | 13.322 | 11.382 | 9.145 | 10.803 | 12.277 | 10.555 | 12.197 | 11.298 | 10.538 | 12.602 | 11.109 |
| TCGA-DK-AA6M | 1582 | 0 | 11.121 | 12.631 | 10.789 | 9.435 | 10.575 | 12.512 | 10.472 | 11.495 | 11.802 | 10.851 | 11.591 | 10.143 |
| TCGA-DK-A1AE | 491 | 0 | 12.087 | 14.009 | 11.942 | 7.629 | 13.720 | 14.055 | 10.854 | 12.184 | 12.785 | 11.844 | 11.666 | 11.001 |
| TCGA-CU-A3KJ | 562 | 0 | 13.531 | 13.909 | 10.414 | 9.651 | 13.098 | 15.159 | 11.875 | 12.006 | 12.338 | 10.921 | 12.036 | 11.348 |
| TCGA-DK-A6AV | 1952 | 0 | 12.316 | 11.900 | 11.362 | 8.238 | 11.372 | 13.505 | 10.831 | 11.988 | 11.867 | 10.883 | 10.791 | 10.187 |
| TCGA-XF-AAML | 232 | 1 | 10.777 | 13.553 | 10.703 | 10.640 | 11.289 | 14.063 | 10.275 | 11.989 | 11.561 | 10.469 | 12.497 | 10.543 |
| TCGA-UY-A8OD | 3432 | 0 | 11.196 | 11.386 | 11.075 | 10.195 | 10.640 | 12.291 | 10.123 | 11.531 | 11.251 | 9.961 | 11.724 | 10.379 |
| TCGA-DK-A1A5 | 65 | 1 | 11.806 | 13.580 | 11.958 | 10.448 | 10.811 | 14.332 | 11.091 | 12.783 | 11.796 | 11.377 | 11.180 | 10.784 |
| TCGA-KQ-A41N | 1604 | 0 | 11.814 | 12.047 | 11.770 | 9.470 | 9.961 | 11.298 | 10.281 | 10.880 | 11.039 | 10.812 | 11.752 | 10.773 |
| TCGA-BT-A0YX | 400 | 1 | 11.167 | 13.024 | 11.084 | 9.496 | 12.337 | 13.570 | 10.159 | 12.001 | 13.515 | 10.078 | 11.307 | 10.103 |
| TCGA-FD-A43X | 110 | 0 | 11.443 | 12.116 | 12.499 | 7.508 | 8.849 | 12.498 | 10.863 | 11.783 | 11.608 | 11.027 | 12.510 | 11.446 |
| TCGA-DK-A6B1 | 2049 | 0 | 11.858 | 13.568 | 11.692 | 10.044 | 11.274 | 13.745 | 11.439 | 12.291 | 12.088 | 10.488 | 12.192 | 11.211 |
| TCGA-E7-A4XJ | 68 | 1 | 10.615 | 11.114 | 11.224 | 9.394 | 10.159 | 10.588 | 9.656 | 10.927 | 10.982 | 9.765 | 10.716 | 10.124 |
| TCGA-FJ-A3ZF | 524 | 0 | 13.461 | 12.879 | 12.262 | 10.477 | 12.350 | 13.908 | 12.099 | 12.922 | 11.370 | 12.323 | 11.797 | 11.914 |
| TCGA-FD-A6TK | 330 | 0 | 11.662 | 13.499 | 10.767 | 11.424 | 12.592 | 13.418 | 11.854 | 12.312 | 12.398 | 11.140 | 12.486 | 10.812 |
| TCGA-G2-A2ES | 1004 | 1 | 12.140 | 13.888 | 11.787 | 9.529 | 13.073 | 13.823 | 10.781 | 11.925 | 12.539 | 11.476 | 11.748 | 11.164 |
| TCGA-DK-A6B6 | 933 | 0 | 11.945 | 11.489 | 11.586 | 9.329 | 9.642 | 12.908 | 10.428 | 11.725 | 11.507 | 10.616 | 11.285 | 9.776 |
| TCGA-CF-A47S | 333 | 0 | 12.204 | 13.238 | 12.730 | 10.067 | 9.504 | 13.278 | 11.692 | 12.248 | 12.101 | 11.489 | 12.789 | 11.854 |
| TCGA-HQ-A2OF | 1947 | 0 | 12.870 | 13.916 | 11.275 | 10.322 | 11.787 | 14.226 | 10.386 | 12.016 | 11.963 | 10.794 | 11.466 | 9.207 |
| TCGA-CF-A27C | 425 | 0 | 12.271 | 11.756 | 12.087 | 6.322 | 10.170 | 12.844 | 10.634 | 11.695 | 11.494 | 11.069 | 11.608 | 10.923 |
| TCGA-H4-A2HQ | 590 | 0 | 12.370 | 14.003 | 10.586 | 11.123 | 11.476 | 14.218 | 10.947 | 11.649 | 12.269 | 10.757 | 11.655 | 10.969 |
| TCGA-BL-A13J | 81 | 1 | 11.291 | 12.956 | 11.115 | 10.627 | 11.602 | 12.710 | 9.432 | 10.212 | 11.202 | 9.462 | 10.718 | 9.409 |
| TCGA-BT-A20J | 579 | 1 | 11.155 | 13.150 | 11.087 | 10.358 | 12.617 | 13.713 | 10.695 | 11.425 | 11.676 | 10.670 | 11.328 | 9.150 |
| TCGA-5N-A9KI | 76 | 1 | 12.581 | 12.990 | 11.318 | 12.620 | 12.650 | 13.527 | 11.649 | 12.412 | 12.927 | 11.058 | 12.498 | 10.611 |
| TCGA-DK-AA6X | 467 | 0 | 10.340 | 12.246 | 10.278 | 8.922 | 9.999 | 12.712 | 9.807 | 11.033 | 11.204 | 9.753 | 10.365 | 9.820 |
| TCGA-XF-A8HH | 57 | 1 | 11.534 | 12.871 | 10.820 | 10.105 | 11.379 | 12.615 | 10.393 | 11.813 | 11.848 | 9.884 | 11.983 | 10.322 |
| TCGA-E7-A7PW | 416 | 0 | 12.812 | 11.295 | 11.027 | 8.647 | 11.763 | 12.024 | 10.429 | 12.293 | 11.036 | 10.886 | 10.905 | 10.961 |
| TCGA-E7-A541 | 529 | 0 | 11.545 | 13.220 | 10.935 | 10.769 | 12.419 | 13.580 | 10.260 | 11.932 | 11.103 | 10.934 | 11.335 | 11.299 |
| TCGA-FD-A6TH | 131 | 1 | 12.967 | 14.341 | 10.585 | 11.593 | 13.312 | 15.273 | 11.827 | 12.887 | 12.287 | 12.043 | 12.326 | 11.140 |
| TCGA-XF-A9T8 | 418 | 1 | 11.650 | 12.949 | 11.530 | 9.484 | 12.637 | 13.009 | 10.359 | 11.502 | 13.219 | 10.661 | 11.525 | 10.364 |
| TCGA-XF-A9ST | 128 | 1 | 12.099 | 14.415 | 10.969 | 11.229 | 12.847 | 15.186 | 11.022 | 12.598 | 12.982 | 10.636 | 11.858 | 10.751 |
| TCGA-DK-A6AW | 1264 | 0 | 10.734 | 11.910 | 10.418 | 9.866 | 10.542 | 11.677 | 9.972 | 10.905 | 10.926 | 10.147 | 11.143 | 10.645 |
| TCGA-K4-A3WV | 646 | 0 | 12.514 | 13.145 | 11.000 | 10.601 | 13.312 | 14.171 | 10.791 | 12.685 | 13.042 | 11.053 | 12.233 | 11.402 |
| TCGA-BT-A3PJ | 390 | 0 | 12.084 | 14.959 | 11.258 | 11.811 | 13.485 | 15.618 | 11.020 | 12.044 | 12.195 | 10.911 | 12.877 | 10.905 |
| TCGA-FJ-A3Z9 | 385 | 1 | 10.965 | 11.191 | 11.113 | 9.961 | 9.313 | 12.132 | 9.656 | 10.656 | 10.887 | 10.312 | 11.485 | 10.162 |
| TCGA-ZF-AA4V | 1806 | 0 | 11.575 | 12.723 | 11.549 | 11.292 | 12.951 | 13.572 | 11.611 | 12.421 | 12.624 | 10.754 | 11.931 | 10.603 |
| TCGA-2F-A9KW | 254 | 1 | 11.860 | 13.991 | 10.799 | 10.256 | 12.311 | 13.015 | 10.663 | 12.309 | 11.769 | 9.941 | 11.964 | 9.999 |
| TCGA-ZF-A9R7 | 665 | 0 | 10.471 | 11.252 | 10.582 | 8.253 | 9.833 | 11.447 | 10.112 | 11.311 | 10.426 | 9.836 | 10.474 | 10.580 |
| TCGA-XF-A9SI | 2423 | 0 | 11.461 | 13.835 | 11.143 | 11.048 | 12.091 | 14.109 | 11.135 | 11.767 | 11.774 | 11.184 | 11.257 | 10.477 |
| TCGA-BT-A20O | 370 | 1 | 11.828 | 13.286 | 10.344 | 10.426 | 12.869 | 13.551 | 10.933 | 11.502 | 11.627 | 10.708 | 11.298 | 10.115 |
| TCGA-GV-A6ZA | 691 | 0 | 11.779 | 13.443 | 11.623 | 8.665 | 11.155 | 13.065 | 10.611 | 11.722 | 11.986 | 11.630 | 12.221 | 10.884 |
| TCGA-XF-AAN2 | 1869 | 1 | 11.105 | 11.403 | 10.142 | 9.426 | 11.214 | 13.594 | 10.136 | 10.974 | 11.087 | 10.003 | 11.114 | 9.474 |
| TCGA-ZF-A9R2 | 642 | 0 | 12.095 | 12.758 | 11.154 | 6.943 | 10.632 | 12.823 | 11.008 | 11.548 | 11.756 | 10.295 | 10.406 | 10.643 |
| TCGA-DK-AA6S | 5050 | 0 | 11.698 | 12.413 | 11.074 | 10.380 | 10.247 | 13.362 | 10.209 | 11.475 | 11.184 | 11.100 | 10.776 | 10.520 |
| TCGA-XF-AAMY | 3011 | 0 | 11.521 | 13.028 | 9.055 | 9.297 | 9.884 | 11.886 | 10.075 | 10.664 | 10.883 | 10.112 | 10.507 | 10.483 |
| TCGA-4Z-AA7Q | 510 | 1 | 11.542 | 13.044 | 10.846 | 10.274 | 11.899 | 13.457 | 10.447 | 12.258 | 12.239 | 10.899 | 11.262 | 10.409 |
| TCGA-C4-A0F0 | 59 | 0 | 11.273 | 12.259 | 10.764 | 10.595 | 12.367 | 12.980 | 10.687 | 11.458 | 12.100 | 8.585 | 12.382 | 8.655 |
| TCGA-XF-A9T3 | 68 | 0 | 10.300 | 11.922 | 9.255 | 10.057 | 10.703 | 11.916 | 9.618 | 10.498 | 10.626 | 9.649 | 11.309 | 9.653 |
| TCGA-BT-A20Q | 593 | 1 | 11.630 | 13.453 | 11.536 | 9.615 | 11.724 | 13.333 | 10.325 | 11.478 | 11.500 | 10.518 | 11.372 | 10.584 |
| TCGA-CF-A3MI | 370 | 0 | 11.504 | 11.464 | 11.631 | 7.492 | 9.364 | 10.000 | 9.841 | 10.740 | 11.216 | 10.280 | 11.693 | 9.901 |
| TCGA-DK-AA74 | 1708 | 0 | 9.745 | 12.124 | 10.214 | 9.735 | 10.371 | 12.150 | 10.161 | 11.228 | 11.860 | 10.247 | 12.510 | 9.954 |
| TCGA-GC-A3WC | 540 | 0 | 11.811 | 13.576 | 10.794 | 7.807 | 12.353 | 12.890 | 10.257 | 11.278 | 12.588 | 10.000 | 11.534 | 10.730 |
| TCGA-SY-A9G5 | 960 | 0 | 12.151 | 12.355 | 11.154 | 10.205 | 10.880 | 12.652 | 11.377 | 12.320 | 11.226 | 10.617 | 11.416 | 10.937 |
| TCGA-4Z-AA87 | 1454 | 0 | 12.893 | 13.445 | 12.332 | 10.604 | 13.860 | 12.942 | 12.940 | 12.039 | 12.061 | 11.649 | 12.046 | 11.821 |
| TCGA-CF-A3MH | 398 | 0 | 11.703 | 12.612 | 11.821 | 9.426 | 10.211 | 12.563 | 10.800 | 11.584 | 11.519 | 10.842 | 11.492 | 10.972 |
| TCGA-XF-A8HB | 1370 | 0 | 12.205 | 13.129 | 11.729 | 9.598 | 12.406 | 13.376 | 11.485 | 12.008 | 12.320 | 10.657 | 12.033 | 10.762 |
| TCGA-UY-A9PD | 542 | 0 | 11.693 | 12.408 | 10.995 | 11.133 | 12.271 | 12.737 | 10.122 | 11.277 | 11.804 | 10.267 | 11.891 | 9.860 |
| TCGA-YF-AA3L | 364 | 0 | 10.931 | 11.823 | 11.740 | 6.304 | 8.647 | 12.093 | 10.987 | 11.717 | 11.320 | 11.561 | 11.962 | 11.296 |
| TCGA-ZF-AA4N | 88 | 1 | 11.208 | 12.973 | 10.248 | 10.296 | 11.626 | 13.679 | 10.383 | 11.123 | 11.649 | 10.570 | 11.803 | 10.116 |
| TCGA-G2-A3IB | 220 | 1 | 12.094 | 12.495 | 11.896 | 9.553 | 12.068 | 13.884 | 11.191 | 11.943 | 11.173 | 10.236 | 12.526 | 11.014 |
| TCGA-ZF-A9R1 | 773 | 0 | 11.259 | 13.726 | 11.185 | 8.349 | 11.350 | 12.757 | 10.652 | 11.781 | 11.759 | 10.581 | 11.076 | 10.788 |
| TCGA-ZF-AA56 | 259 | 1 | 11.688 | 13.714 | 11.406 | 9.669 | 11.700 | 13.999 | 10.821 | 11.990 | 12.734 | 10.278 | 11.729 | 10.498 |
| TCGA-S5-A6DX | 56 | 1 | 11.679 | 11.184 | 12.022 | 9.644 | 10.512 | 10.600 | 11.025 | 11.622 | 12.804 | 10.574 | 12.999 | 10.712 |
| TCGA-DK-AA6W | 415 | 1 | 12.240 | 13.960 | 10.917 | 8.629 | 13.026 | 13.869 | 11.147 | 12.320 | 12.168 | 10.584 | 10.747 | 11.135 |
| TCGA-CF-A47Y | 373 | 0 | 10.817 | 11.906 | 11.361 | 7.943 | 8.690 | 11.409 | 10.136 | 10.856 | 11.324 | 10.683 | 11.438 | 10.704 |
| TCGA-FD-A43P | 454 | 0 | 13.122 | 14.299 | 11.912 | 9.555 | 12.207 | 14.249 | 11.611 | 12.691 | 12.176 | 12.706 | 12.309 | 11.410 |
| TCGA-XF-AAN7 | 565 | 1 | 10.316 | 11.972 | 9.704 | 9.464 | 11.182 | 12.591 | 9.562 | 10.469 | 10.961 | 10.033 | 10.617 | 9.875 |
| TCGA-FD-A3NA | 1338 | 0 | 12.150 | 14.053 | 11.473 | 11.713 | 12.431 | 13.864 | 11.102 | 12.639 | 12.187 | 10.913 | 12.174 | 10.776 |
| TCGA-GU-AATP | 1003 | 0 | 12.183 | 12.123 | 11.099 | 10.951 | 11.357 | 11.735 | 10.537 | 11.031 | 11.024 | 11.279 | 11.526 | 9.984 |
| TCGA-ZF-A9R0 | 680 | 1 | 11.240 | 12.032 | 11.353 | 9.262 | 9.944 | 12.097 | 9.655 | 11.485 | 10.845 | 9.974 | 11.287 | 10.722 |
| TCGA-GV-A3JX | 581 | 0 | 12.675 | 14.437 | 10.304 | 10.781 | 12.645 | 14.128 | 11.018 | 12.668 | 11.928 | 12.023 | 12.104 | 11.512 |
| TCGA-FD-A3N6 | 483 | 0 | 12.181 | 12.927 | 11.362 | 8.276 | 12.060 | 13.287 | 10.651 | 11.675 | 11.837 | 10.890 | 11.785 | 10.803 |
| TCGA-XF-A9T4 | 495 | 1 | 11.370 | 14.321 | 11.458 | 11.200 | 11.295 | 14.342 | 11.042 | 11.998 | 12.307 | 11.331 | 12.290 | 11.212 |
| TCGA-2F-A9KQ | 2787 | 0 | 11.623 | 13.615 | 11.076 | 8.690 | 10.609 | 13.474 | 10.452 | 11.393 | 11.024 | 10.138 | 11.295 | 10.254 |
| TCGA-4Z-AA7S | 1064 | 1 | 11.754 | 12.361 | 11.015 | 8.006 | 11.067 | 12.457 | 10.700 | 11.252 | 11.254 | 10.609 | 11.101 | 10.730 |
| TCGA-E7-A677 | 443 | 0 | 11.735 | 13.507 | 12.066 | 10.334 | 11.487 | 14.141 | 11.234 | 12.048 | 12.357 | 11.293 | 12.616 | 11.458 |
| TCGA-K4-A4AC | 278 | 1 | 11.344 | 12.414 | 11.370 | 8.778 | 11.743 | 13.153 | 10.607 | 11.786 | 11.769 | 10.384 | 12.783 | 10.136 |
| TCGA-FD-A6TD | 386 | 1 | 11.251 | 13.420 | 11.050 | 9.238 | 11.689 | 13.627 | 10.990 | 12.110 | 12.391 | 10.715 | 12.095 | 11.331 |
| TCGA-FT-A3EE | 99 | 1 | 12.472 | 14.326 | 11.767 | 12.777 | 13.393 | 15.436 | 11.142 | 13.411 | 12.957 | 11.762 | 12.650 | 10.390 |
| TCGA-DK-A2HX | 55 | 0 | 13.210 | 13.912 | 11.749 | 11.775 | 12.113 | 13.439 | 11.811 | 11.686 | 12.485 | 11.419 | 12.701 | 10.778 |
| TCGA-ZF-A9RN | 615 | 1 | 11.444 | 13.685 | 10.487 | 5.700 | 13.001 | 15.105 | 10.918 | 12.395 | 11.535 | 10.908 | 10.940 | 10.943 |
| TCGA-BT-A20P | 544 | 1 | 10.434 | 12.262 | 11.020 | 7.833 | 9.803 | 11.172 | 10.040 | 11.132 | 10.870 | 10.014 | 10.776 | 10.211 |
| TCGA-DK-A3WX | 321 | 1 | 11.649 | 14.699 | 11.936 | 10.877 | 12.259 | 14.170 | 11.056 | 11.989 | 11.868 | 10.781 | 12.377 | 10.160 |
| TCGA-ZF-A9RM | 1455 | 0 | 11.760 | 13.567 | 11.331 | 9.853 | 11.071 | 13.269 | 10.728 | 11.577 | 11.871 | 11.362 | 11.832 | 10.647 |
| TCGA-FD-A6TF | 69 | 1 | 11.846 | 13.222 | 11.317 | 11.355 | 13.033 | 13.699 | 10.544 | 12.375 | 11.674 | 11.505 | 12.539 | 10.712 |
| TCGA-DK-A2I6 | 2656 | 0 | 11.813 | 14.747 | 12.064 | 11.104 | 14.342 | 14.957 | 12.575 | 12.725 | 12.940 | 12.818 | 13.019 | 11.603 |
| TCGA-ZF-AA54 | 590 | 1 | 12.216 | 13.863 | 10.752 | 9.555 | 13.890 | 13.857 | 10.935 | 11.961 | 12.377 | 10.685 | 11.890 | 10.128 |
| TCGA-CF-A47W | 368 | 0 | 10.990 | 11.867 | 12.430 | 6.989 | 8.267 | 12.101 | 11.101 | 11.427 | 12.068 | 10.792 | 12.168 | 11.138 |
| TCGA-G2-AA3F | 893 | 0 | 12.244 | 13.596 | 11.983 | 11.853 | 12.358 | 13.797 | 11.389 | 12.073 | 12.276 | 11.536 | 12.187 | 10.866 |
| TCGA-C4-A0EZ | 273 | 1 | 11.777 | 13.324 | 10.609 | 7.672 | 14.285 | 14.163 | 10.726 | 12.228 | 11.711 | 10.984 | 12.055 | 11.233 |
| TCGA-4Z-AA86 | 311 | 1 | 12.001 | 14.238 | 11.542 | 11.087 | 12.348 | 14.292 | 11.143 | 12.374 | 11.968 | 11.622 | 12.165 | 10.798 |
| TCGA-XF-AAN1 | 941 | 1 | 10.842 | 11.366 | 11.693 | 7.219 | 9.267 | 12.213 | 10.040 | 11.206 | 11.254 | 10.317 | 11.041 | 9.231 |
| TCGA-FD-A5BS | 1261 | 0 | 9.989 | 12.377 | 10.501 | 10.563 | 11.456 | 12.786 | 9.976 | 11.513 | 11.641 | 10.341 | 11.530 | 10.091 |
| TCGA-E5-A2PC | 1326 | 0 | 11.930 | 13.569 | 12.031 | 10.022 | 11.125 | 14.568 | 11.338 | 12.236 | 13.090 | 11.100 | 12.374 | 11.308 |
| TCGA-CU-A0YR | 460 | 1 | 11.457 | 12.838 | 10.658 | 10.006 | 12.086 | 13.136 | 9.961 | 11.156 | 12.128 | 9.780 | 12.019 | 9.602 |
| TCGA-G2-AA3D | 1960 | 0 | 11.296 | 14.439 | 11.177 | 9.757 | 10.757 | 13.089 | 10.876 | 11.591 | 12.061 | 10.450 | 11.534 | 10.890 |
| TCGA-UY-A78K | 536 | 1 | 12.655 | 12.624 | 11.205 | 9.841 | 12.408 | 13.030 | 11.077 | 12.553 | 11.078 | 11.516 | 11.816 | 11.334 |
| TCGA-GU-A42Q | 344 | 1 | 11.948 | 13.631 | 11.214 | 9.480 | 12.698 | 14.321 | 10.351 | 11.696 | 12.218 | 10.028 | 11.417 | 11.408 |
| TCGA-GC-A3YS | 758 | 0 | 13.054 | 12.848 | 10.830 | 10.486 | 11.726 | 13.667 | 12.009 | 11.627 | 11.752 | 10.334 | 11.326 | 11.451 |
| TCGA-ZF-AA4X | 2044 | 0 | 11.679 | 12.581 | 11.305 | 10.278 | 11.229 | 12.852 | 10.861 | 11.719 | 11.674 | 11.305 | 11.798 | 10.492 |
| TCGA-XF-A9SM | 1048 | 0 | 11.038 | 13.148 | 10.679 | 9.725 | 12.103 | 14.068 | 10.751 | 11.867 | 11.833 | 10.732 | 11.722 | 10.665 |
| TCGA-HQ-A2OE | 1174 | 0 | 12.243 | 13.614 | 11.816 | 11.331 | 12.089 | 15.228 | 11.260 | 13.519 | 13.098 | 12.226 | 12.073 | 11.579 |
| TCGA-YC-A89H | 573 | 0 | 11.181 | 12.689 | 11.709 | 10.441 | 11.394 | 12.642 | 10.424 | 11.600 | 11.938 | 10.403 | 11.708 | 10.530 |
| TCGA-ZF-A9RL | 2703 | 0 | 11.944 | 12.413 | 11.214 | 5.883 | 10.840 | 12.258 | 11.000 | 11.271 | 11.241 | 10.943 | 10.932 | 11.053 |
| TCGA-E7-A97P | 437 | 1 | 11.167 | 12.684 | 11.128 | 9.778 | 11.793 | 13.552 | 10.431 | 11.440 | 11.796 | 10.326 | 11.217 | 10.415 |
| TCGA-FD-A6TB | 572 | 0 | 12.385 | 13.845 | 11.212 | 9.785 | 12.268 | 13.858 | 11.117 | 12.055 | 11.693 | 11.770 | 12.230 | 11.048 |
| TCGA-GC-A3RD | 428 | 0 | 11.927 | 13.892 | 11.767 | 11.220 | 10.824 | 13.213 | 10.722 | 11.994 | 12.153 | 11.002 | 11.666 | 11.051 |
| TCGA-FD-A5BR | 656 | 0 | 11.111 | 13.578 | 11.362 | 9.833 | 10.856 | 12.946 | 10.146 | 11.372 | 11.424 | 10.532 | 12.472 | 10.883 |
| TCGA-ZF-A9RE | 106 | 1 | 11.775 | 13.281 | 11.315 | 7.687 | 12.058 | 13.315 | 10.050 | 11.253 | 12.042 | 9.937 | 11.309 | 10.519 |
| TCGA-UY-A9PB | 899 | 0 | 11.208 | 13.058 | 10.485 | 10.264 | 12.011 | 13.414 | 10.837 | 11.756 | 11.485 | 11.388 | 11.943 | 10.456 |
| TCGA-DK-A3WW | 497 | 0 | 11.344 | 12.481 | 11.673 | 8.480 | 12.366 | 13.694 | 10.525 | 11.903 | 11.920 | 10.737 | 11.662 | 10.578 |
| TCGA-2F-A9KT | 2352 | 0 | 11.586 | 12.325 | 10.964 | 10.350 | 10.939 | 12.095 | 10.083 | 12.342 | 11.426 | 10.728 | 11.851 | 10.927 |
| TCGA-LT-A8JT | 641 | 0 | 11.364 | 13.363 | 11.581 | 9.264 | 10.741 | 13.400 | 10.621 | 11.758 | 11.739 | 10.615 | 11.560 | 10.696 |
| TCGA-FD-A3B4 | 477 | 0 | 12.570 | 13.245 | 11.746 | 10.237 | 12.273 | 13.513 | 11.198 | 11.744 | 12.200 | 11.496 | 12.283 | 11.174 |
| TCGA-FD-A5BT | 328 | 1 | 9.811 | 12.147 | 10.870 | 11.258 | 10.312 | 12.170 | 10.313 | 11.860 | 12.289 | 10.869 | 13.175 | 10.746 |
| TCGA-DK-A3WY | 4283 | 0 | 11.109 | 13.357 | 11.118 | 9.510 | 12.355 | 13.614 | 11.695 | 12.120 | 11.970 | 11.370 | 11.485 | 11.036 |
| TCGA-R3-A69X | 433 | 0 | 11.780 | 14.434 | 10.859 | 10.797 | 11.565 | 14.107 | 11.202 | 11.883 | 12.427 | 11.140 | 12.398 | 10.616 |
| TCGA-G2-A3VY | 536 | 0 | 12.999 | 14.430 | 11.442 | 12.289 | 12.416 | 14.645 | 12.944 | 13.296 | 12.392 | 12.074 | 12.132 | 11.793 |
| TCGA-K4-A5RJ | 53 | 0 | 11.720 | 12.845 | 10.536 | 7.889 | 11.561 | 13.619 | 11.475 | 11.788 | 11.799 | 10.813 | 11.734 | 10.291 |
| TCGA-BL-A5ZZ | 377 | 0 | 11.016 | 13.736 | 10.845 | 12.289 | 12.235 | 14.160 | 10.320 | 11.985 | 12.283 | 10.786 | 12.231 | 10.665 |
| TCGA-E5-A4TZ | 467 | 1 | 12.616 | 13.374 | 12.166 | 9.545 | 12.581 | 12.890 | 11.242 | 12.694 | 13.128 | 11.727 | 11.542 | 11.933 |
| TCGA-4Z-AA89 | 1029 | 0 | 10.992 | 12.460 | 11.354 | 7.531 | 9.649 | 12.326 | 10.004 | 11.025 | 11.226 | 10.206 | 11.008 | 10.246 |
| TCGA-G2-A2EK | 485 | 0 | 11.531 | 13.612 | 11.554 | 10.843 | 10.081 | 14.039 | 10.744 | 11.626 | 12.257 | 10.498 | 11.536 | 10.422 |
| TCGA-MV-A51V | 410 | 0 | 11.688 | 12.618 | 9.901 | 10.679 | 10.409 | 12.728 | 10.766 | 11.611 | 11.455 | 10.299 | 11.717 | 10.516 |
| TCGA-C4-A0F6 | 700 | 0 | 11.358 | 12.338 | 10.736 | 11.771 | 11.256 | 13.330 | 9.723 | 11.273 | 10.803 | 10.186 | 10.937 | 8.945 |
| TCGA-K4-A54R | 842 | 0 | 10.957 | 12.519 | 10.417 | 11.851 | 11.354 | 13.045 | 10.203 | 11.922 | 11.720 | 10.358 | 12.148 | 10.628 |
| TCGA-G2-A2EO | 1072 | 0 | 13.202 | 12.600 | 11.740 | 9.923 | 13.474 | 12.679 | 11.304 | 11.808 | 12.089 | 10.963 | 12.091 | 10.473 |
| TCGA-DK-A3IT | 459 | 0 | 12.185 | 14.067 | 11.423 | 10.269 | 12.216 | 13.854 | 10.782 | 12.271 | 12.197 | 10.863 | 12.528 | 10.345 |
| TCGA-XF-A9SW | 362 | 1 | 10.584 | 10.712 | 9.276 | 9.702 | 10.977 | 11.438 | 9.596 | 10.634 | 10.651 | 9.607 | 10.057 | 9.137 |
| TCGA-ZF-AA5H | 897 | 0 | 12.445 | 13.030 | 11.188 | 11.355 | 12.581 | 14.349 | 11.063 | 11.775 | 11.947 | 11.194 | 12.717 | 11.265 |
| TCGA-FD-A3SP | 401 | 0 | 10.994 | 14.054 | 10.358 | 11.044 | 12.016 | 13.714 | 10.469 | 11.794 | 11.786 | 11.045 | 11.956 | 10.791 |
| TCGA-K4-A6FZ | 55 | 0 | 12.401 | 13.614 | 12.260 | 10.120 | 12.255 | 13.757 | 10.917 | 12.088 | 12.040 | 10.691 | 11.965 | 10.995 |
| TCGA-FD-A5C0 | 407 | 0 | 12.531 | 12.955 | 11.071 | 9.234 | 11.762 | 13.267 | 11.018 | 12.583 | 11.705 | 10.885 | 10.994 | 11.263 |
| TCGA-FD-A5BZ | 835 | 1 | 11.011 | 13.118 | 10.594 | 11.184 | 11.217 | 12.960 | 10.701 | 11.769 | 11.175 | 10.042 | 11.816 | 10.380 |
| TCGA-DK-A6B2 | 477 | 0 | 12.218 | 14.418 | 10.464 | 10.551 | 11.763 | 14.124 | 10.929 | 12.387 | 12.573 | 10.428 | 11.862 | 10.914 |
| TCGA-DK-A1AD | 3420 | 0 | 11.319 | 13.207 | 11.799 | 11.870 | 10.863 | 14.476 | 12.510 | 11.884 | 12.254 | 11.499 | 11.754 | 11.219 |
| TCGA-E7-A7DU | 28 | 0 | 11.107 | 11.259 | 11.079 | 6.700 | 8.150 | 11.471 | 9.776 | 10.477 | 10.453 | 10.171 | 10.729 | 10.431 |
| TCGA-DK-A2I1 | 546 | 0 | 12.041 | 13.794 | 12.066 | 11.835 | 11.784 | 13.748 | 11.011 | 12.094 | 12.709 | 10.567 | 12.656 | 10.688 |
| TCGA-E7-A519 | 508 | 0 | 9.669 | 8.960 | 11.285 | 7.577 | 7.672 | 8.492 | 9.726 | 10.772 | 11.127 | 10.415 | 11.271 | 10.458 |
| TCGA-UY-A9PA | 1072 | 0 | 10.796 | 12.800 | 10.925 | 8.011 | 9.771 | 12.799 | 10.146 | 10.619 | 10.855 | 10.137 | 10.834 | 9.963 |
| TCGA-GU-A42R | 344 | 0 | 12.512 | 12.797 | 11.526 | 8.344 | 10.863 | 13.398 | 11.560 | 11.570 | 11.695 | 10.679 | 11.073 | 10.868 |
| TCGA-UY-A9PF | 117 | 0 | 11.075 | 12.852 | 10.013 | 7.948 | 9.187 | 11.717 | 9.317 | 9.996 | 10.712 | 9.042 | 11.964 | 9.790 |
| TCGA-E7-A8O7 | 466 | 0 | 11.820 | 13.842 | 11.645 | 9.231 | 9.428 | 13.559 | 10.877 | 11.611 | 11.412 | 11.068 | 11.644 | 10.734 |
| TCGA-DK-A1AF | 536 | 0 | 11.720 | 14.058 | 11.528 | 11.672 | 11.844 | 14.080 | 10.724 | 11.907 | 12.347 | 10.860 | 12.500 | 10.874 |
| TCGA-4Z-AA7Y | 1494 | 0 | 11.474 | 13.336 | 11.206 | 6.000 | 10.724 | 13.370 | 10.428 | 11.018 | 10.590 | 10.358 | 10.984 | 10.577 |
| TCGA-GV-A3QI | 322 | 0 | 13.871 | 13.580 | 11.544 | 12.458 | 13.824 | 14.422 | 10.603 | 12.469 | 13.513 | 12.782 | 11.974 | 10.461 |
| TCGA-ZF-AA4R | 1036 | 1 | 11.690 | 12.948 | 10.534 | 10.649 | 11.680 | 13.721 | 10.399 | 11.904 | 11.687 | 10.742 | 10.967 | 10.502 |
| TCGA-XF-A9SH | 1971 | 1 | 10.776 | 11.607 | 10.538 | 6.129 | 9.605 | 11.669 | 9.124 | 10.162 | 10.784 | 9.736 | 10.393 | 9.518 |
| TCGA-4Z-AA82 | 1556 | 1 | 11.949 | 13.702 | 10.077 | 10.194 | 12.250 | 14.053 | 11.097 | 11.982 | 11.739 | 9.587 | 11.441 | 10.115 |
| TCGA-CF-A9FM | 398 | 0 | 11.226 | 12.765 | 11.334 | 6.492 | 9.003 | 12.520 | 10.288 | 11.274 | 11.350 | 10.066 | 11.157 | 10.394 |
| TCGA-K4-A6MB | 469 | 0 | 11.945 | 13.797 | 11.669 | 11.356 | 12.208 | 13.920 | 12.099 | 12.368 | 12.490 | 10.929 | 12.081 | 11.327 |
| TCGA-HQ-A5ND | 274 | 1 | 11.987 | 13.838 | 11.513 | 8.814 | 11.862 | 14.431 | 10.974 | 11.444 | 12.191 | 10.125 | 11.402 | 10.194 |
| TCGA-FJ-A3ZE | 324 | 1 | 12.223 | 12.227 | 12.087 | 11.105 | 11.246 | 11.849 | 10.588 | 10.997 | 12.396 | 11.438 | 12.098 | 10.637 |
| TCGA-CU-A3QU | 158 | 0 | 12.390 | 13.653 | 11.592 | 10.877 | 11.360 | 13.292 | 11.512 | 12.232 | 11.968 | 11.165 | 12.045 | 11.300 |
| TCGA-K4-A3WU | 105 | 0 | 12.317 | 13.590 | 11.668 | 11.274 | 11.990 | 13.220 | 11.354 | 12.423 | 12.419 | 11.765 | 12.076 | 11.004 |
| TCGA-DK-A3IM | 238 | 0 | 12.536 | 12.665 | 11.206 | 9.353 | 12.896 | 13.696 | 11.133 | 12.273 | 12.452 | 10.902 | 11.897 | 11.389 |
| TCGA-DK-AA77 | 618 | 0 | 12.039 | 13.876 | 11.301 | 10.051 | 12.785 | 13.578 | 10.659 | 11.559 | 11.733 | 10.445 | 11.564 | 10.308 |
| TCGA-GD-A3OP | 64 | 0 | 11.914 | 13.163 | 11.472 | 9.839 | 10.735 | 13.744 | 11.377 | 12.150 | 11.603 | 11.501 | 11.883 | 11.107 |
| TCGA-XF-AAN8 | 118 | 1 | 9.869 | 10.856 | 10.071 | 10.592 | 11.289 | 11.396 | 8.977 | 11.021 | 11.311 | 9.714 | 10.843 | 9.870 |
| TCGA-GV-A40E | 261 | 1 | 11.676 | 12.789 | 11.015 | 13.937 | 13.291 | 14.050 | 11.257 | 12.268 | 12.526 | 11.155 | 12.281 | 11.177 |
| TCGA-XF-A8HG | 467 | 1 | 11.229 | 12.187 | 10.776 | 7.531 | 10.331 | 13.310 | 9.993 | 11.421 | 10.958 | 10.324 | 10.967 | 9.655 |
| TCGA-ZF-A9R5 | 1090 | 0 | 10.785 | 12.499 | 11.697 | 7.651 | 9.317 | 12.509 | 10.570 | 11.474 | 11.751 | 10.609 | 11.350 | 10.540 |
| TCGA-CF-A8HX | 345 | 0 | 11.043 | 9.883 | 10.978 | 7.087 | 8.745 | 9.382 | 10.180 | 11.099 | 10.817 | 10.906 | 10.352 | 11.242 |
| TCGA-S5-AA26 | 503 | 0 | 11.096 | 12.135 | 10.530 | 7.033 | 8.833 | 10.943 | 9.741 | 10.658 | 10.314 | 10.376 | 10.451 | 10.519 |
| TCGA-XF-A8HF | 2954 | 1 | 11.397 | 12.820 | 11.677 | 11.698 | 11.237 | 12.873 | 11.015 | 11.872 | 11.823 | 11.134 | 12.530 | 10.247 |
| TCGA-E7-A85H | 394 | 0 | 13.666 | 13.520 | 11.960 | 11.958 | 12.701 | 13.070 | 11.618 | 12.011 | 12.555 | 11.414 | 12.165 | 12.311 |
| TCGA-XF-A9SL | 2020 | 1 | 10.928 | 12.301 | 10.816 | 10.917 | 10.841 | 12.481 | 10.445 | 11.448 | 11.862 | 10.445 | 11.841 | 10.534 |
| TCGA-DK-AA71 | 415 | 0 | 11.512 | 12.271 | 11.245 | 7.735 | 10.586 | 12.053 | 10.621 | 10.910 | 11.241 | 10.530 | 11.285 | 10.256 |
| TCGA-DK-AA6T | 572 | 0 | 10.541 | 11.288 | 10.467 | 7.200 | 12.718 | 12.984 | 11.214 | 12.516 | 10.806 | 10.283 | 10.409 | 11.698 |
| TCGA-GV-A3QK | 832 | 0 | 11.825 | 12.156 | 11.754 | 7.877 | 10.892 | 13.164 | 10.864 | 12.189 | 11.964 | 11.674 | 12.532 | 11.023 |
| TCGA-DK-A3IK | 67 | 0 | 11.849 | 12.645 | 11.914 | 11.179 | 10.490 | 12.217 | 10.867 | 12.073 | 12.513 | 10.533 | 11.441 | 11.087 |
| TCGA-DK-A2I2 | 237 | 1 | 10.999 | 13.547 | 10.890 | 10.074 | 12.118 | 14.368 | 10.756 | 12.084 | 12.348 | 10.377 | 12.801 | 10.292 |
| TCGA-G2-A3IE | 612 | 1 | 12.647 | 12.948 | 11.755 | 10.093 | 10.179 | 12.774 | 11.015 | 11.721 | 11.460 | 11.942 | 11.679 | 10.834 |
| TCGA-GU-AATQ | 213 | 1 | 10.882 | 11.734 | 10.526 | 7.937 | 11.266 | 12.321 | 10.075 | 10.582 | 12.139 | 9.662 | 10.615 | 9.878 |
| TCGA-K4-A83P | 495 | 0 | 12.097 | 13.138 | 11.050 | 9.615 | 11.493 | 13.423 | 10.643 | 11.892 | 11.657 | 10.594 | 11.273 | 11.040 |
| TCGA-GV-A3QF | 617 | 1 | 12.373 | 14.276 | 10.235 | 12.091 | 11.656 | 13.446 | 11.726 | 12.231 | 12.114 | 10.707 | 12.222 | 11.190 |
| TCGA-CF-A47V | 379 | 0 | 11.328 | 11.977 | 11.322 | 7.322 | 8.830 | 11.397 | 10.321 | 11.517 | 10.540 | 10.660 | 11.328 | 11.428 |
| TCGA-FD-A3SM | 547 | 1 | 11.237 | 14.048 | 11.444 | 10.472 | 11.282 | 13.648 | 10.709 | 11.855 | 11.814 | 10.578 | 11.353 | 10.688 |
| TCGA-GU-A766 | 480 | 0 | 11.962 | 13.667 | 11.733 | 8.823 | 12.990 | 13.669 | 11.200 | 11.917 | 12.509 | 11.316 | 12.501 | 10.798 |
| TCGA-XF-AAMH | 344 | 1 | 10.831 | 13.747 | 11.670 | 10.505 | 11.552 | 14.295 | 10.483 | 13.296 | 12.087 | 10.461 | 13.272 | 10.944 |
| TCGA-GC-A4ZW | 15 | 0 | 11.526 | 14.082 | 11.028 | 9.362 | 12.590 | 13.367 | 10.509 | 11.814 | 12.414 | 11.375 | 11.355 | 11.661 |
| TCGA-GD-A6C6 | 67 | 0 | 10.950 | 10.433 | 12.285 | 9.142 | 10.141 | 11.778 | 10.956 | 11.499 | 13.027 | 12.403 | 12.526 | 11.706 |
| TCGA-HQ-A5NE | 370 | 1 | 11.882 | 13.574 | 10.953 | 10.428 | 11.426 | 13.951 | 11.011 | 12.241 | 11.632 | 10.694 | 11.966 | 10.315 |
| TCGA-CU-A0YN | 393 | 1 | 11.743 | 12.998 | 10.962 | 10.043 | 11.737 | 13.502 | 9.996 | 11.680 | 11.784 | 10.564 | 12.028 | 10.990 |
| TCGA-CF-A8HY | 345 | 0 | 10.964 | 11.198 | 12.238 | 7.895 | 9.177 | 12.331 | 10.403 | 11.351 | 11.775 | 10.785 | 12.182 | 10.672 |
| TCGA-DK-A3X1 | 1460 | 0 | 13.068 | 12.132 | 11.903 | 11.353 | 11.815 | 12.490 | 10.895 | 11.303 | 11.870 | 10.226 | 11.251 | 10.437 |
| TCGA-XF-A9SK | 486 | 1 | 11.211 | 12.809 | 10.514 | 10.656 | 10.077 | 13.087 | 10.855 | 12.270 | 11.562 | 10.937 | 11.659 | 10.222 |
| TCGA-UY-A9PE | 189 | 0 | 10.863 | 12.087 | 10.554 | 10.448 | 10.607 | 11.955 | 10.031 | 11.521 | 10.900 | 10.875 | 11.168 | 9.963 |
| TCGA-FD-A62P | 191 | 1 | 11.681 | 14.037 | 11.003 | 11.389 | 11.982 | 13.491 | 10.978 | 12.704 | 12.622 | 11.276 | 12.356 | 10.583 |
| TCGA-GU-A764 | 610 | 0 | 11.582 | 12.841 | 11.283 | 11.787 | 12.145 | 14.272 | 10.897 | 12.635 | 12.524 | 10.701 | 12.319 | 10.846 |
| TCGA-2F-A9KO | 678 | 0 | 11.526 | 12.677 | 10.962 | 9.543 | 11.370 | 13.282 | 9.844 | 11.092 | 11.336 | 11.045 | 11.093 | 10.093 |
| TCGA-FD-A3SQ | 1423 | 1 | 10.885 | 13.552 | 11.018 | 10.877 | 10.700 | 13.700 | 10.507 | 11.910 | 11.900 | 10.798 | 11.970 | 10.839 |
| TCGA-FD-A6TA | 1912 | 0 | 11.177 | 12.764 | 11.273 | 9.950 | 11.188 | 13.487 | 10.388 | 11.515 | 11.926 | 9.913 | 11.734 | 10.709 |
| TCGA-ZF-AA53 | 1761 | 0 | 11.724 | 12.934 | 10.824 | 9.217 | 11.895 | 13.465 | 10.264 | 11.481 | 11.797 | 10.380 | 11.580 | 10.864 |
| TCGA-XF-AAMR | 2790 | 0 | 12.485 | 14.174 | 12.293 | 11.225 | 12.203 | 14.204 | 11.622 | 12.454 | 12.346 | 11.198 | 12.027 | 11.729 |
| TCGA-E7-A4IJ | 408 | 0 | 9.786 | 13.292 | 11.156 | 11.447 | 10.484 | 12.989 | 12.797 | 11.893 | 11.782 | 10.197 | 11.539 | 11.114 |
| TCGA-BT-A42C | 873 | 0 | 12.376 | 13.192 | 11.662 | 9.315 | 11.696 | 13.749 | 12.061 | 12.250 | 12.517 | 11.183 | 11.956 | 11.423 |
| TCGA-4Z-AA83 | 1943 | 0 | 10.626 | 12.571 | 10.794 | 6.409 | 9.377 | 12.474 | 9.984 | 11.107 | 10.762 | 9.964 | 11.274 | 10.786 |
| TCGA-XF-AAMW | 253 | 1 | 10.552 | 12.102 | 10.161 | 9.877 | 10.848 | 13.108 | 9.822 | 10.753 | 11.226 | 9.602 | 11.626 | 9.723 |
| TCGA-G2-A2EC | 696 | 1 | 11.504 | 12.701 | 11.521 | 10.678 | 10.131 | 13.406 | 10.727 | 11.428 | 11.916 | 10.492 | 11.652 | 10.133 |
| TCGA-CF-A1HR | 389 | 0 | 11.448 | 13.628 | 11.539 | 7.788 | 10.568 | 13.027 | 10.464 | 11.599 | 12.351 | 11.254 | 11.488 | 9.638 |
| TCGA-FD-A3SL | 683 | 0 | 11.788 | 13.013 | 11.229 | 10.133 | 11.051 | 12.814 | 11.071 | 11.509 | 11.931 | 10.902 | 12.101 | 10.906 |
| TCGA-BT-A20U | 455 | 1 | 10.803 | 13.044 | 10.605 | 8.672 | 11.441 | 13.057 | 9.451 | 11.023 | 11.272 | 9.999 | 10.749 | 9.849 |
| TCGA-ZF-A9RD | 408 | 1 | 10.644 | 10.949 | 10.596 | 7.384 | 10.645 | 12.163 | 10.186 | 10.664 | 11.442 | 10.475 | 11.930 | 9.502 |
| TCGA-DK-AA6Q | 413 | 1 | 11.988 | 12.888 | 11.633 | 8.435 | 11.554 | 13.193 | 10.342 | 11.152 | 11.793 | 10.237 | 11.289 | 9.587 |
| TCGA-DK-A2I4 | 3835 | 0 | 11.813 | 14.546 | 11.698 | 10.881 | 12.521 | 14.542 | 11.189 | 12.075 | 12.195 | 11.128 | 11.692 | 11.045 |
| TCGA-FD-A3B7 | 122 | 1 | 11.040 | 13.688 | 10.514 | 11.441 | 12.394 | 14.431 | 10.661 | 11.741 | 12.001 | 10.660 | 13.047 | 10.905 |
| TCGA-E7-A7DV | 37 | 0 | 11.174 | 14.176 | 11.410 | 12.528 | 12.161 | 14.724 | 12.813 | 12.200 | 12.512 | 12.271 | 13.058 | 11.177 |
| TCGA-ZF-AA5N | 168 | 1 | 10.155 | 11.423 | 9.858 | 11.228 | 11.051 | 12.256 | 9.207 | 10.940 | 11.414 | 8.811 | 11.659 | 8.655 |
| TCGA-DK-A1AG | 475 | 0 | 11.564 | 13.336 | 12.017 | 8.607 | 10.578 | 12.865 | 10.697 | 11.924 | 11.809 | 11.081 | 11.923 | 11.141 |
| TCGA-CU-A3YL | 496 | 0 | 11.506 | 13.283 | 11.355 | 7.592 | 9.823 | 11.561 | 10.828 | 11.979 | 11.940 | 11.447 | 11.948 | 11.195 |
| TCGA-LC-A66R | 466 | 0 | 10.494 | 11.442 | 9.420 | 8.714 | 11.610 | 12.531 | 11.175 | 10.549 | 10.632 | 9.736 | 11.099 | 9.748 |
| TCGA-GV-A40G | 428 | 0 | 12.469 | 12.778 | 11.609 | 10.566 | 11.716 | 12.994 | 11.773 | 12.276 | 12.043 | 11.772 | 11.762 | 11.988 |
| TCGA-FD-A3B8 | 378 | 0 | 11.517 | 14.252 | 10.443 | 10.776 | 12.106 | 14.332 | 13.162 | 11.736 | 12.114 | 10.979 | 11.578 | 10.926 |
| TCGA-BT-A3PK | 303 | 1 | 12.218 | 14.500 | 12.199 | 11.868 | 13.089 | 14.731 | 11.107 | 12.401 | 13.178 | 12.139 | 12.683 | 11.524 |
| TCGA-XF-A9T0 | 799 | 0 | 11.780 | 13.485 | 11.438 | 11.249 | 10.693 | 13.085 | 10.465 | 12.327 | 11.853 | 10.943 | 11.580 | 10.664 |
| TCGA-BL-A3JM | 205 | 1 | 12.716 | 14.878 | 11.122 | 9.518 | 13.725 | 14.674 | 11.627 | 13.119 | 13.343 | 11.764 | 11.848 | 11.091 |
| TCGA-PQ-A6FN | 507 | 0 | 10.912 | 12.479 | 10.948 | 9.353 | 12.066 | 13.704 | 10.905 | 12.372 | 12.997 | 11.274 | 13.203 | 10.849 |
| TCGA-4Z-AA81 | 1270 | 1 | 11.873 | 11.828 | 10.208 | 10.800 | 11.512 | 13.034 | 10.956 | 11.575 | 11.261 | 9.875 | 11.366 | 9.768 |
| TCGA-C4-A0F1 | 89 | 0 | 10.781 | 12.091 | 10.896 | 8.524 | 10.441 | 13.102 | 10.122 | 11.111 | 12.282 | 10.089 | 11.380 | 10.102 |
| TCGA-DK-A6B0 | 2330 | 0 | 11.048 | 12.807 | 12.121 | 7.170 | 9.373 | 12.726 | 10.778 | 11.737 | 11.739 | 10.957 | 12.172 | 11.052 |
| TCGA-G2-A2EF | 1884 | 0 | 12.337 | 14.615 | 11.564 | 10.728 | 12.915 | 14.400 | 12.103 | 12.883 | 12.676 | 11.867 | 12.898 | 11.836 |
| TCGA-2F-A9KP | 364 | 1 | 11.737 | 14.232 | 11.410 | 8.362 | 11.360 | 13.268 | 10.386 | 11.978 | 11.622 | 11.355 | 11.626 | 11.013 |
| TCGA-ZF-AA5P | 372 | 0 | 11.165 | 12.837 | 10.670 | 9.472 | 11.472 | 13.409 | 10.667 | 11.589 | 11.751 | 10.645 | 11.343 | 10.987 |
| TCGA-E7-A5KE | 17 | 0 | 12.066 | 12.466 | 11.305 | 9.238 | 10.707 | 13.239 | 11.326 | 11.492 | 12.105 | 10.850 | 11.992 | 10.914 |
| TCGA-K4-AAQO | 359 | 0 | 10.596 | 12.260 | 10.798 | 9.384 | 10.590 | 13.435 | 13.390 | 11.566 | 11.080 | 10.242 | 11.343 | 10.329 |
| TCGA-CF-A9FF | 361 | 0 | 11.089 | 11.634 | 10.706 | 7.562 | 9.482 | 10.842 | 9.662 | 10.657 | 10.533 | 9.781 | 10.699 | 10.456 |
| TCGA-CF-A7I0 | 368 | 0 | 11.350 | 11.823 | 10.878 | 7.700 | 9.889 | 11.832 | 10.157 | 11.482 | 10.938 | 10.194 | 10.672 | 10.431 |
| TCGA-SY-A9G0 | 378 | 0 | 10.827 | 12.564 | 9.844 | 10.174 | 10.588 | 12.660 | 9.547 | 11.093 | 11.097 | 10.152 | 11.053 | 10.032 |
| TCGA-CU-A0YO | 149 | 1 | 11.132 | 12.710 | 11.067 | 10.439 | 10.211 | 12.851 | 10.017 | 10.701 | 11.856 | 10.535 | 11.536 | 10.061 |
| TCGA-XF-A9T5 | 2027 | 0 | 11.222 | 12.239 | 10.690 | 9.638 | 12.112 | 13.480 | 10.156 | 11.813 | 12.342 | 10.470 | 11.057 | 10.032 |
| TCGA-K4-A5RI | 356 | 1 | 10.929 | 12.995 | 10.576 | 9.000 | 11.956 | 13.821 | 10.569 | 11.766 | 12.190 | 10.094 | 11.470 | 10.548 |
| TCGA-FD-A3B6 | 209 | 0 | 11.663 | 14.486 | 11.598 | 10.994 | 13.096 | 14.367 | 11.122 | 12.444 | 12.980 | 11.659 | 12.647 | 11.027 |
| TCGA-KQ-A41Q | 361 | 0 | 12.513 | 12.469 | 11.842 | 9.482 | 10.907 | 12.825 | 11.177 | 11.717 | 11.829 | 10.434 | 11.828 | 10.575 |
| TCGA-XF-A9T6 | 64 | 0 | 10.281 | 11.513 | 11.629 | 9.557 | 12.485 | 12.591 | 10.244 | 11.293 | 11.979 | 10.801 | 12.879 | 10.520 |
| TCGA-FD-A5C1 | 1603 | 0 | 10.942 | 12.392 | 10.841 | 10.456 | 12.077 | 13.333 | 10.743 | 11.985 | 11.295 | 10.678 | 12.109 | 10.147 |
| TCGA-E7-A3Y1 | 163 | 0 | 10.920 | 11.502 | 11.427 | 7.238 | 8.758 | 10.696 | 10.119 | 11.322 | 11.362 | 10.574 | 11.343 | 10.581 |
| TCGA-BT-A2LB | 492 | 1 | 12.492 | 13.382 | 11.402 | 10.943 | 10.930 | 14.129 | 11.190 | 12.492 | 11.202 | 11.477 | 12.121 | 11.127 |
| TCGA-FD-A62N | 82 | 0 | 10.883 | 12.644 | 10.700 | 9.470 | 11.941 | 13.420 | 10.895 | 11.782 | 11.834 | 11.150 | 12.732 | 10.633 |
| TCGA-BT-A20R | 154 | 1 | 11.058 | 13.551 | 10.772 | 12.247 | 12.034 | 13.878 | 10.832 | 12.193 | 11.911 | 10.132 | 12.167 | 10.669 |
| TCGA-UY-A78P | 2380 | 0 | 11.695 | 12.357 | 11.791 | 9.022 | 12.200 | 13.048 | 10.744 | 11.379 | 11.988 | 10.790 | 11.920 | 10.089 |
| TCGA-FD-A62S | 368 | 0 | 11.543 | 13.382 | 10.404 | 11.228 | 11.964 | 13.819 | 10.791 | 11.875 | 11.858 | 10.612 | 12.725 | 11.003 |
| TCGA-XF-A9SZ | 859 | 1 | 12.935 | 13.312 | 11.167 | 10.845 | 12.217 | 13.063 | 11.852 | 11.787 | 12.664 | 10.586 | 12.394 | 10.531 |
| TCGA-ZF-AA52 | 1077 | 1 | 11.068 | 12.016 | 10.564 | 10.220 | 10.899 | 12.372 | 10.439 | 11.291 | 11.678 | 11.448 | 11.918 | 11.416 |
| TCGA-ZF-A9RC | 2868 | 0 | 11.312 | 12.053 | 10.736 | 10.098 | 10.328 | 12.204 | 10.296 | 11.553 | 11.322 | 11.767 | 11.175 | 11.127 |
| TCGA-KQ-A41P | 1094 | 0 | 11.823 | 12.779 | 11.180 | 10.564 | 11.160 | 12.516 | 13.048 | 11.889 | 12.075 | 12.341 | 12.175 | 10.445 |
| TCGA-G2-AA3C | 211 | 1 | 10.307 | 12.670 | 11.605 | 9.834 | 11.100 | 13.157 | 11.079 | 11.891 | 12.311 | 10.815 | 12.254 | 10.863 |
| TCGA-XF-A9SP | 454 | 1 | 11.508 | 12.380 | 10.468 | 10.706 | 10.987 | 13.226 | 11.109 | 12.020 | 11.528 | 10.638 | 12.133 | 10.765 |
| TCGA-DK-AA6L | 1163 | 1 | 11.159 | 13.523 | 11.023 | 10.780 | 10.700 | 13.530 | 11.109 | 12.228 | 12.264 | 10.960 | 11.677 | 11.554 |
| TCGA-FD-A3N5 | 363 | 0 | 12.560 | 13.673 | 11.551 | 10.155 | 13.034 | 14.084 | 10.948 | 12.209 | 12.552 | 10.468 | 11.843 | 11.116 |
| TCGA-XF-AAMG | 3364 | 0 | 11.499 | 12.866 | 10.920 | 11.501 | 12.218 | 13.852 | 10.806 | 12.658 | 11.554 | 9.344 | 11.316 | 11.405 |
| TCGA-GU-A763 | 824 | 0 | 11.196 | 12.714 | 12.813 | 7.714 | 10.755 | 13.219 | 11.407 | 12.368 | 12.585 | 11.293 | 12.711 | 10.981 |
| TCGA-DK-A3IV | 294 | 1 | 12.039 | 13.482 | 12.024 | 10.599 | 11.308 | 12.526 | 11.455 | 11.643 | 12.201 | 11.190 | 12.347 | 11.553 |
| TCGA-4Z-AA80 | 19 | 1 | 13.109 | 13.259 | 10.951 | 6.426 | 12.373 | 12.959 | 11.805 | 11.710 | 10.803 | 10.928 | 11.292 | 10.521 |
| TCGA-BT-A2LD | 623 | 1 | 12.004 | 13.192 | 10.467 | 11.103 | 12.551 | 14.048 | 14.836 | 12.271 | 11.649 | 10.671 | 11.685 | 10.266 |
| TCGA-ZF-A9R3 | 949 | 1 | 11.480 | 13.401 | 11.826 | 9.373 | 10.807 | 13.827 | 10.928 | 12.350 | 11.576 | 11.005 | 11.753 | 10.653 |
| TCGA-FJ-A871 | 272 | 1 | 11.042 | 13.733 | 12.061 | 11.372 | 11.379 | 14.254 | 11.295 | 12.392 | 11.594 | 11.770 | 12.624 | 11.760 |
| TCGA-YC-A9TC | 20 | 1 | 11.575 | 13.274 | 11.437 | 8.322 | 13.157 | 13.078 | 10.815 | 12.030 | 11.961 | 10.752 | 11.746 | 10.463 |
| TCGA-UY-A8OB | 2109 | 0 | 11.519 | 12.753 | 11.897 | 9.570 | 12.069 | 14.167 | 11.024 | 12.654 | 11.982 | 10.370 | 11.743 | 10.241 |
| TCGA-XF-A9SV | 388 | 1 | 10.950 | 11.297 | 10.662 | 9.109 | 9.646 | 11.053 | 10.054 | 9.970 | 10.767 | 9.455 | 10.218 | 9.543 |
| TCGA-DK-A1A7 | 560 | 0 | 11.854 | 11.793 | 11.910 | 8.919 | 10.719 | 11.398 | 12.753 | 11.177 | 12.019 | 11.535 | 11.448 | 11.480 |
| TCGA-GU-A767 | 144 | 1 | 11.769 | 12.707 | 11.868 | 10.920 | 10.901 | 12.949 | 11.191 | 11.759 | 11.993 | 10.348 | 12.749 | 11.059 |
| TCGA-DK-A3IS | 1529 | 0 | 11.997 | 13.059 | 12.468 | 10.313 | 11.292 | 13.310 | 11.198 | 11.614 | 12.470 | 11.390 | 12.916 | 11.147 |
| TCGA-CF-A5UA | 365 | 0 | 11.687 | 11.558 | 12.527 | 8.508 | 10.042 | 11.449 | 11.423 | 11.099 | 10.909 | 11.384 | 11.818 | 10.801 |
| TCGA-XF-AAMQ | 2177 | 0 | 12.730 | 13.455 | 11.610 | 9.905 | 11.811 | 13.973 | 11.613 | 11.980 | 12.205 | 11.575 | 12.016 | 11.232 |
| TCGA-BT-A20W | 254 | 1 | 11.087 | 12.814 | 10.679 | 9.562 | 10.922 | 12.877 | 10.809 | 11.027 | 10.945 | 10.650 | 10.669 | 10.058 |
| TCGA-DK-A1AA | 578 | 0 | 11.781 | 14.099 | 11.896 | 8.820 | 11.006 | 14.081 | 10.910 | 11.674 | 12.107 | 10.502 | 11.978 | 10.686 |
| TCGA-XF-AAMT | 90 | 1 | 11.103 | 13.199 | 10.353 | 10.646 | 11.367 | 14.169 | 10.644 | 11.515 | 12.355 | 10.387 | 12.584 | 10.929 |
| TCGA-FD-A3SO | 168 | 1 | 11.004 | 13.062 | 11.516 | 10.147 | 11.908 | 13.776 | 9.579 | 11.716 | 11.867 | 11.386 | 12.008 | 9.830 |
| TCGA-CF-A47T | 205 | 0 | 11.230 | 11.677 | 11.771 | 8.340 | 10.148 | 11.309 | 10.224 | 11.271 | 11.471 | 9.484 | 12.178 | 10.508 |
| TCGA-ZF-AA51 | 1714 | 0 | 11.326 | 12.765 | 10.603 | 10.592 | 10.849 | 13.407 | 9.984 | 12.014 | 10.851 | 11.384 | 10.929 | 10.574 |
| TCGA-E7-A6ME | 395 | 0 | 11.496 | 13.349 | 11.631 | 9.979 | 11.687 | 13.521 | 10.947 | 12.334 | 11.240 | 10.857 | 11.348 | 10.887 |
| TCGA-FD-A43U | 454 | 0 | 11.232 | 12.081 | 12.540 | 9.760 | 10.814 | 12.104 | 10.857 | 11.818 | 11.936 | 11.285 | 12.319 | 11.226 |
| TCGA-XF-A8HC | 200 | 1 | 11.375 | 11.731 | 10.493 | 8.531 | 9.329 | 11.790 | 11.005 | 11.346 | 11.905 | 11.109 | 11.599 | 10.444 |
| TCGA-CF-A5U8 | 399 | 0 | 10.879 | 11.902 | 11.563 | 8.124 | 8.581 | 12.251 | 10.488 | 11.266 | 11.623 | 10.738 | 11.279 | 10.663 |
| TCGA-UY-A78N | 2641 | 1 | 11.766 | 12.485 | 11.357 | 9.858 | 11.836 | 13.164 | 10.809 | 11.978 | 11.486 | 11.564 | 11.870 | 11.141 |
| TCGA-XF-A9SX | 719 | 1 | 10.741 | 12.313 | 10.354 | 10.328 | 11.026 | 12.491 | 10.434 | 11.411 | 11.164 | 11.071 | 12.004 | 10.333 |
| TCGA-DK-A3IQ | 539 | 1 | 10.488 | 12.470 | 10.521 | 11.844 | 10.536 | 12.939 | 10.054 | 11.231 | 11.767 | 10.725 | 11.877 | 10.649 |
| TCGA-XF-A8HE | 3817 | 0 | 11.896 | 12.241 | 11.279 | 8.707 | 11.204 | 13.222 | 10.180 | 11.811 | 12.026 | 11.104 | 11.291 | 10.725 |

Supplementary Table 2. Parameters of Multivariate Cox Regression

| id | coef | HR | HR.95L | | HR.95H | pvalue |
| --- | --- | --- | --- | --- | --- | --- |
| CEBPG | -0.20011 | 0.818638 | 0.583887 | 1.147769 | | 0.245793 |
| HYOU1 | 0.244704 | 1.277243 | 0.933123 | 1.748269 | | 0.126561 |
| IMP3 | -0.24457 | 0.783045 | 0.550797 | 1.113223 | | 0.173059 |
| KDELR3 | -0.00178 | 0.998217 | 0.864208 | 1.153006 | | 0.980644 |
| MTHFD2 | 0.103924 | 1.109516 | 0.874217 | 1.408147 | | 0.392791 |
| PDIA6 | 0.050603 | 1.051905 | 0.736854 | 1.501659 | | 0.780537 |
| POP4 | -0.57326 | 0.563686 | 0.384267 | 0.826878 | | 0.003364 |
| PREB | 0.300137 | 1.350043 | 0.902921 | 2.018579 | | 0.143634 |
| SRPRB | 0.195614 | 1.216057 | 0.813811 | 1.817124 | | 0.339792 |
| TATDN2 | -0.21746 | 0.804562 | 0.576553 | 1.12274 | | 0.200888 |
| YIF1A | 0.453465 | 1.573756 | 1.087631 | 2.277158 | | 0.016147 |
| ZBTB17 | -0.47078 | 0.624516 | 0.435107 | 0.896379 | | 0.010672 |
